# Roles of chromosomal and gonadal sex in the fetal and placental responses to maternal food restriction in mice

**DOI:** 10.1093/molehr/gaaf015

**Published:** 2025-04-26

**Authors:** Jess C Hercus, Daniel Alejandro Salcedo Rubio, Maria Elisa Osorio Nieto, Cheayeong Keum, Qi Wang, John A Macdonald, Jordan S Scott, Emily R J Lucas, Julian K Christians

**Affiliations:** Department of Biological Sciences, Simon Fraser University, Burnaby, BC, Canada; Department of Biological Sciences, Simon Fraser University, Burnaby, BC, Canada; Department of Biological Sciences, Simon Fraser University, Burnaby, BC, Canada; Department of Biological Sciences, Simon Fraser University, Burnaby, BC, Canada; Department of Biological Sciences, Simon Fraser University, Burnaby, BC, Canada; Department of Biological Sciences, Simon Fraser University, Burnaby, BC, Canada; Department of Biological Sciences, Simon Fraser University, Burnaby, BC, Canada; Department of Biological Sciences, Simon Fraser University, Burnaby, BC, Canada; Department of Biological Sciences, Simon Fraser University, Burnaby, BC, Canada; Centre for Cell Biology, Development and Disease, Simon Fraser University, Burnaby, BC, Canada; British Columbia Children’s Hospital Research Institute, Vancouver, BC, Canada; Women’s Health Research Institute, BC Women’s Hospital and Health Centre, Vancouver, BC, Canada

**Keywords:** placenta, RNA sequencing, transcriptomics, sex differences, malnutrition, labyrinth, junctional zone, decidua

## Abstract

It is hypothesized that male fetuses are more vulnerable to *in utero* insults than females due to different growth strategies, and that the placenta contributes to these sex differences. We examined sex differences in the fetal and placental responses to maternal food restriction (∼60% of *ad libitum*) beginning mid-gestation (Day 11.5). To dissect the roles of chromosomal and gonadal sex, we used the Four Core Genotypes mouse model, which combines deletion of the testis-determining *Sry* gene from the Y chromosome and autosomal insertion of the *Sry* gene, such that XX gonadal males and XY gonadal females are produced in addition to XX females and XY males. Food restriction reduced fetal and placental weights but had no effect on the number of viable conceptuses. However, this effect did not differ between gonadal male and female, or between XX and XY, conceptuses. Sex differences in gene expression in both the labyrinth and the combined junctional zone/decidua, as assessed by RNA sequencing, were due entirely to chromosomal sex and not gonadal sex. Food restriction affected the expression of 525 and 665 genes in the labyrinth and the junctional zone/decidua, respectively. However, these effects of food restriction did not differ by gonadal or chromosomal sex when assessed for statistical interactions. In contrast, when analyzing XX and XY placentas separately, hundreds of genes were affected by food restriction in one sex but not in the other, including hundreds of genes not found to be significant in the combined analyses. However, estimated effect sizes were generally similar for XX and XY placentas, suggesting that these sex-stratified analyses greatly exaggerated the extent of sex-dependent responses. Overall, we did not find evidence of the hypothesized sex differences in fetal growth strategy and found that sex differences in placental gene expression were largely due to chromosomal sex.

## Graphical Abstract

**Graphical Abstract:**
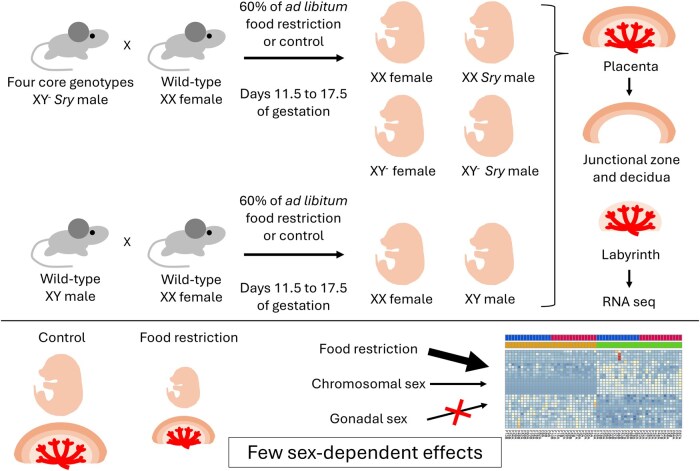
Maternal food restriction in the Four Core Genotypes model; the testis-determining *Sry* gene is on an autosome and deleted on the Y-chromosome (Y^−^), allowing chromosomal sex to be studied independently of gonadal status.

## Introduction

Male fetuses are on average heavier than female fetuses beginning in the first trimester (Catalano *et al.*, 1995; Bukowski *et al.*, 2007; Broere-Brown *et al.*, 2016; Kiserud *et al.*, 2017), but also have higher risks of adverse gestational and perinatal outcomes (Stevenson *et al.*, 2000; Challis *et al.*, 2013; Mondal *et al.*, 2014; Verburg *et al.*, 2016; Al-Qaraghouli and Fang, 2017; Broere-Brown *et al.*, 2020; Voskamp *et al.*, 2020). Many authors have suggested that there is a causal link between these two observations, whereby males prioritize growth at the expense of placental development, resulting in increased mortality and susceptibility to prenatal insults, whereas females are more responsive to challenges, reducing growth in the presence of adversity, and thereby reducing mortality and increasing resilience (Clifton, 2010; Eriksson *et al.*, 2010; Sandman *et al.*, 2013; Bale, 2016; Devaskar and Chu, 2016; DiPietro and Voegtline, 2017; Nugent *et al.*, 2018; Pérez-Cerezales *et al.*, 2018; Sutherland and Brunwasser, 2018; Kozhemiako *et al.*, 2020; Meakin *et al.*, 2021).

Such hypothesized sex differences are likely mediated, at least in part by the placenta, which shares the chromosomal complement of the fetus and thus has a sex (Clifton, 2010; Gabory *et al.*, 2013; Rosenfeld, 2015; Tarrade *et al.*, 2015; Bronson and Bale, 2016; Burton *et al.*, 2016; Cheong *et al.*, 2016; Kalisch-Smith *et al.*, 2017; Dearden *et al.*, 2018; Pérez-Cerezales *et al.*, 2018; Meakin *et al.*, 2021; Christians, 2022). However, the mechanisms underlying sex differences in the response to, and long-term effects of, early-life environment are not well known (Christians and Reue, 2023). In mammals, sex differences may be due to chromosomal sex (i.e. XX or XY chromosomes), and/or gonadal sex (i.e. presence of ovaries or testes) with the associated production of sex steroids. In mice, it is possible to distinguish between effects of chromosomal sex and gonadal sex using the Four Core Genotypes (FCG) model (Burgoyne and Arnold, 2016; Mauvais-Jarvis *et al.*, 2017). This model combines a deletion in the testis-determining *Sry* gene on the Y chromosome (referred to as Y^−^), with an insertion of the *Sry* gene on an autosome (Arnold, 2020; Reue and Wiese, 2022). When an XY^−^  *Sry* male is mated with a wild-type XX female, the resulting offspring have four potential genotypes (the FCG): XX *Sry* males (with testes), XY^−^ females (with ovaries), as well as normal XX females and XY^−^  *Sry* males (carrying *Sry* on an autosome rather than the Y chromosome). Gonadal sex is thereby independent of the sex chromosome complement. The FCG model has been used extensively to study sex differences in adults but has rarely been applied to prenatal or placental traits (but see Ishikawa *et al.*, 2003).

The goals of the present work were to: (i) assess whether there are sex differences in fetal and placental responses to food restriction, to test the hypothesis that males prioritize fetal growth at the expense of placental development, whereas females are better able to respond to prenatal insults (Meakin *et al.*, 2021); and (ii) use the FCG mouse model to assess the relative contributions of chromosomal complement and fetal gonads to (A) the response to food restriction and (B) sex differences in placental gene expression. In addition to FCG mice, we also included wild-type matings to assess to what extent differences between XX females and XY^−^  *Sry* males reflect differences between wild-type females and males. As in humans, fetal and placental weights in rodents are generally greater in males than in females (Vickers *et al.*, 2011; Gao *et al.*, 2012; Chin *et al.*, 2017; Christians *et al.*, 2018b; Denisova *et al.*, 2020; Eaton *et al.*, 2020; Phuthong *et al.*, 2020; Bidne *et al.*, 2021). Moreover, while human pregnancies are typically singleton, mouse pregnancies carry multiple fetuses of mixed sex but each fetus is almost always associated with its own placenta, enabling placental function to differ by sex in both cases. As a model of prenatal adversity, we used food restriction beginning in mid-gestation, based on the hypothesis that males ‘invest less in placental growth, which puts them at greater risk of becoming undernourished’ (Eriksson *et al.*, 2010). Furthermore, this timing is common among rodent studies, and food restriction beginning in mid-gestation has relatively consistent sex-dependent effects on adult blood pressure of offspring (Christians *et al.*, 2021). In mice, the differentiation of the gonads occurs between Days 12 and 14 (Wolstenholme *et al.*, 2013), and given the presence of androgen and estrogen receptors within the placenta (McWhorter *et al.*, 2018; Deegan and Engel, 2019; Meakin and Clifton, 2019), both chromosomal complement and fetal gonadal sex may influence the placental response to food restriction occurring in the latter half of gestation. The observations that male fetuses adjacent to females *in utero* influence the anogenital distance (AGD) of the females (Hotchkiss and Vandenbergh, 2005), and that female fetuses adjacent to males influence the adult behavior of the males (vom Saal *et al.*, 1983), further support the plausibility that gonadal sex may influence tissues beyond the fetus.

## Materials and methods

### Animals

All work was carried out in accordance with the guidelines of the Canadian Council on Animal Care and approved by the SFU University Animal Care Committee (protocol 1332B-21). Male and female C57BL/6J and FCG mice were purchased from the Jackson Laboratory (Bar Harbor, ME, USA, stock numbers 000664 and 010905, respectively). Mice were housed with a maximum of five mice per cage with water and food (5001 LabDiet, St Louis, MO, USA) available *ad libitum*. Prior to their first mating, females were switched to a breeder diet (Prolab RMH 3000, LabDiet) *ad libitum*. Female C57BL/6J mice were weighed and placed with a single-housed male (C57BL/6J or FCG) overnight with a maximum of two females per male and separated from the male the next morning (gestational day 0.5). Matings to C57BL/6J and FCG males were carried out using the same cohort of females in the same facility at the same time. Sample sizes are provided in Table 1; we mated more females to FCG males than to C57BL/6J males to obtain roughly similar numbers of fetuses of each genotype (i.e. females mated to FCG males have fetuses of four genotypes, whereas females mated to C57BL/6J males have fetuses of the two wild-type sexes). Pregnancy was determined at Day 11.5 based on weight gain ≥2.5 g. On gestational day 11.5, pregnant females were single-housed and randomly allocated to either the *ad libitum* diet (control) group, or the food restriction group, which received ∼3.5 g of breeder diet daily. Food consumption was monitored daily; food restriction females had always consumed all food by the following day. Females that were not pregnant at Day 11.5 were paired again. At cull, females ranged in age from 12 to 22 weeks (median 15 weeks) and had been mated one to six (median three) times to achieve pregnancy. The number of pregnancies in each group is provided in Table 1.

**Table 1.** Sample sizes.

Treatment	Mating type	Females	Conceptuses	XXF	XYM	XXM	XYF
**Total**							
Food restriction	**FCG**	18	146	36	40	37	32
	**Wild type**	11	76	45	31	–	–
Control	**FCG**	17^a^	137	31	37	32	37
	**Wild type**	10	82	36	46	–	–
**RNA sequencing**							
Food restriction	**FCG**	18	20	5	5	5	5^b^
	**Wild type**	10	10	5	5	–	–
Control	**FCG**	16	20	5	5	5	5^b^
	**Wild type**	10	10	5	5	–	–

FCG, four core genotypes; XXF, gonadal female with XX chromosomes; XXM, gonadal male with XX chromosomes; XYF, gonadal female with XY chromosomes; XYM, gonadal male with XY chromosomes.

a In one control FCG female, fetuses appeared normal but were very small (average <0.2 g compared to >0.45 g in next lightest litter); these were excluded from all analyses of fetal and placental traits.

b Due to removal of outliers (described in text), sample sizes were reduced to four control XYF and four food restriction XYF in the labyrinth, and three control XYF in the junctional zone/decidua.

### Sample collection

Mice were culled by cervical dislocation at gestational day 17.5 within 1 min of moving their home cage. The uterus was immediately removed and placed in PBS on ice. The number of viable-appearing conceptuses, and the number of resorbed conceptuses (small, dark masses) were counted. The first two placentas closest to the ovary in one horn of the uterus were immediately dissected in PBS under a digital microscope to separate the labyrinth from the junctional zone and decidua (i.e. junctional zone and decidua were combined), and then the two regions were separately snap frozen in liquid nitrogen. Expression of canonical cell-type markers indicated good tissue separation (Hercus *et al.*, 2024b). Snap-frozen samples were stored at −80°C. The remainder of the uterus was placed in 10% neutral buffered formalin solution at 4°C for 2 days before dissection to weigh the remaining fetuses and placentas. Fetuses were imaged using a digital microscope to measure AGD, crown-rump length, and the length of the head (measured from the tip of the nose through the middle of the eye to the back of the head). A subset of fetuses (two different genotypes from each dam and balanced with regards to treatment and genotype) were selected for dissection to measure kidney size to assess whether this might be associated with effects of prenatal food restriction on offspring adult blood pressure (Christians *et al.*, 2021). Fetal kidneys were imaged using a digital microscope to obtain the 2D areas of the kidney in profile. Researchers were blind to genotype and treatment during fetal measurements. Fetal tail clips were collected for DNA extraction. During dissection of conceptuses for histology, the placental interface with the uterus was kept intact, and placentas were stored in 70% ethanol at 4°C prior to paraffin embedding and sectioning by the Histology Core at the BC Children’s Hospital Research Institute. We analyzed three sections per placenta, taken ∼100 µm apart, beginning at the center of the placenta.

### Genotyping

Genomic DNA was extracted from tail clips. For FCG matings, fetal genotype was determined by PCR using the method recommended by the Jackson Laboratory (Protocol 5990). Primers used for the amplification of the Tg(Sry)2Ei transgene were Jax 9371 forward primer: 5′-AGC CCT ACA GCC ACA TGA TA-3′; Jax 9372 reverse primer: 5′-GTC TTG CCT GTA TGT GAT GG-3′; primers used for the detection of the Y chromosome mutation were Jax 9369 forward primer: 5′-CTG GAG CTC TAC AGT GAT GA-3′; Jax 9370 reverse primer: 5′-CAG TTA CCA ATC AAC ACA TCA C-3′); and an internal positive control from our own lab comprised of KO_dist2: 5′-CTC TTG CAT GCC TCC ACT AC-3′; KO_exon2: 5′-GGT CAA ATG AAA CTT CCC TCC-3′). For wild-type matings, fetal sex was also determined by PCR (McFarlane *et al.*, 2013).

### RNA extraction

The number of samples selected for RNA extraction and sequencing from each group is provided in Table 1. In all cases, we extracted and sequenced both the labyrinth and junctional zone/decidua from a given placenta, i.e. labyrinth samples and junctional zone/decidua samples are matched. Where possible, we used only one placenta from each dam, but to balance sample sizes it was necessary to use two placentas of different genotypes from each of six dams.

We homogenized placental tissue using zirconium oxide beads (0.5 mm, ZROB05, Next Advance, Troy, NY, USA) and a mechanical shaker (MM300, Retsch, Haan, Germany). Placental mRNA was extracted using the Qiagen RNeasy Plus kit (Qiagen, Hilden, Germany), including gDNA Eliminator columns to remove genomic DNA, according to manufacturer instructions. We assessed RNA concentrations using a Nanodrop spectrophotometer (ND-2000C, ThermoFisher Scientific, Waltham, MA, USA), and RNA was sent to the UBC School of Biomedical Engineering (SBME) Sequencing Core for quality control, library preparation, and sequencing. Sample quality control was performed using the Agilent 2100 Bioanalyzer or the Agilent 4200 Tapestation (Agilent, Santa Clara, CA, USA) and RNA quality was high for all tissues; the median RIN was 9.4 and 9.4, and the lowest RIN value was 8.3 and 7.9 for the labyrinth and junctional zone/decidua, respectively. Samples were then prepped following the standard protocol for the Illumina Stranded mRNA prep (Illumina, San Diego, CA, USA). Sequencing was performed on the Illumina NextSeq2000 with Paired-End 59 bp×59 bp reads, with 20 million paired-end reads per sample. Read sequences were aligned to the Mus musculus (mm10) reference sequence using DRAGEN RNA app on Basespace Sequence Hub. For all samples, over 85% of sequences mapped to transcript fragments (median 92% and 90% for the labyrinth and junctional zone/decidua, respectively), reflecting low gDNA contamination. Sequencing and RNA metrics are provided in Supplementary Tables S1 and S2. Sequencing data are deposited in the GEO database under accession numbers GSE285453 (labyrinth) and GSE291544 (junctional zone/decidua).

### Data analysis: pregnancy outcomes and fetal data

All statistical analyses were performed using general linear models (proc GLM), non-parametric Wilcoxon two-sample tests (for the number of fetuses and resorbed conceptuses, proc NPAR1WAY), or repeated measures analyses (proc MIXED) in SAS, Version 9.4 (SAS Institute Inc., Cary, NC, USA). Repeated measures analyses (with dam as a random factor) were used for placental and fetal traits where there were multiple offspring per dam, since the dam was the unit of replication. Specific models are described below.

### Data analysis: RNAseq

Gene expression analyses were performed in R (version 4.4.0). Raw count data were extracted from Salmon files using tximport (Soneson *et al.*, 2016), then analyzed with DESeq2 (Love *et al.*, 2014). To remove genes with low expression, we pre-filtered to retain only genes that had a count of at least 10 in at least 10 samples. Analyses were done separately on labyrinth, and junctional zone/decidua. DESeq2 model designs are described below. One sample (F6A, a control XY female) associated with an abnormally small fetus was removed from transcriptomic analyses. In addition, one sample was removed in each of the labyrinth (F41B, a food restriction XY female) and junctional zone/decidua (F46B, a control XY female) based on unusual expression patterns for many genes as assessed by heatmaps. We used a Benjamini–Hochberg false discovery rate-adjusted *P*-value cutoff of 0.05 to determine significance. Gene enrichment analyses were performed using g: Profiler (Kolberg *et al.*, 2023), using all genes that remained after pre-filtering as the background. Figures were produced using ggplot2 (Wickham, 2016) and VennDiagram (Chen and Boutros, 2011).

## Results

Sample sizes are provided in Table 1. Food restriction females were provided with a constant amount of food each day, but control females varied slightly in their daily consumption, such that the amount of food provided to food restriction females was 55, 60, 64, 56, 59, and 66% of that consumed by control females on Days 11.5, 12.5, 13.5, 14.5, 15.5, and 16.5, respectively.

### Female traits and pregnancy outcomes

**Figure 1.**
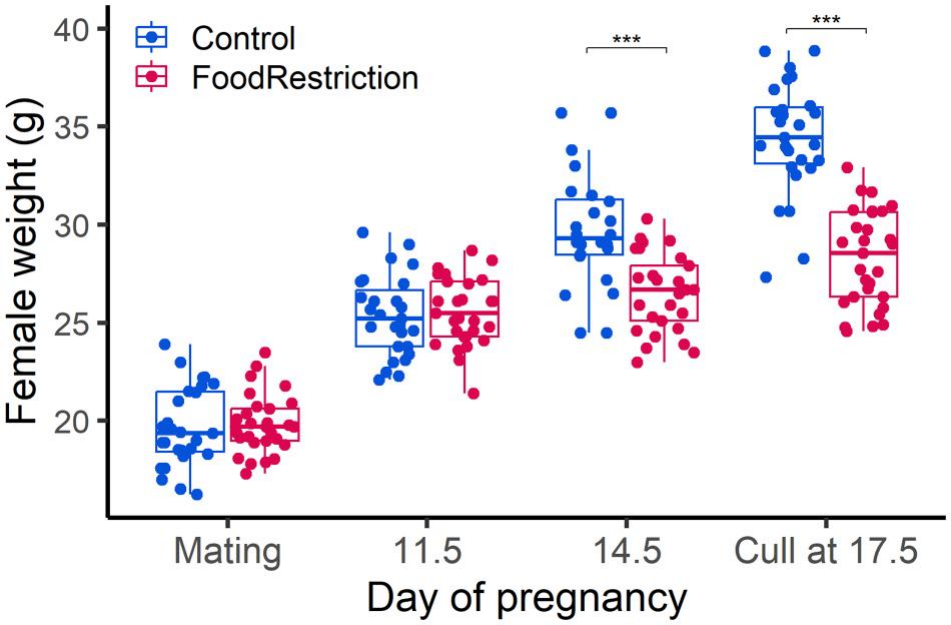
**Female weight at mating and through pregnancy in control and food restriction females.** Blue symbols: control; Red symbols: food restriction. ***P < 0.0001.

All females identified as pregnant on Day 11.5 based on weight gain were pregnant at cull at Day 17.5, and no females deemed not pregnant were later found to be pregnant. At mating and at Day 11.5, i.e. prior to the food restriction, female weight did not differ between treatment groups (Fig. 1). However, at Day 14.5, 3 days after food restriction had started, food restriction females were significantly lighter (Fig. 1). At cull, food restriction females were ∼6 g lighter, with a ∼4 g difference in the weight of the female without uterus and conceptuses, and ∼2 g difference in the total weight of the uterus and conceptuses (Fig. 1, Table 2). Between mating and cull, control females had gained ∼4.6 g not including the uterus and conceptuses, whereas food-restricted females had not gained significant weight (Table 2). There was no difference in female age between groups (Table 2).

**Table 2.** Female traits and pregnancy outcomes.

Trait	Control (means±SE)	Food restriction (means±SE)	**Difference** ^a^ (means±SE)
**Female age at cull (weeks)**	15.4 ± 0.5	15.8 ± 0.5	−0.4 ± 0.7
**Female without uterus and conceptuses (g)**	24.3 ± 0.4	20.1 ± 0.3	4.2 ± 0.5***
**Weight of the uterus and conceptuses (g)**	10.1 ± 0.4	8.2 ± 0.4	1.9 ± 0.6**
**Female weight gain between mating and cull (not including uterus and conceptuses) (g)**	4.6 ± 0.2	0.2 ± 0.2	4.4 ± 0.3***
**Number of viable-appearing conceptuses**	8.1 ± 0.4	7.6 ± 0.4	0.5 ± 0.5
**Number of resorbed conceptuses**	0.7 ± 0.2	0.9 ± 0.2	−0.2 ± 0.3

a Difference between groups is significant at *P* < 0.01 (**) or *P* < 0.0001 (***).

The difference in total conceptus weight was not due to a difference in the number of fetuses that appeared viable at dissection (food restriction mean: 7.6, control mean: 8.1, *P* = 0.61) nor a difference in the number of resorbed conceptuses (food restriction mean: 0.9, control mean: 0.7, *P* = 0.35). There was no difference between FCG and wild-type matings in the number of fetuses that appeared viable at dissection (FCG mean: 8.1, wild-type mean: 7.5, *P* = 0.74), or in the number of resorbed conceptuses (FCG mean: 0.8, wild-type mean: 0.9, *P* = 0.79).

### Fetal outcomes

Among fetuses from matings to FCG males, the ratios of the four genotypes did not differ from the expected 1:1:1:1, the ratio of gonadal females to gonadal males did not differ from the expected 1:1, and the ratio of XX to XY did not differ from the expected 1:1 (Table 3). Furthermore, each of these ratios did not differ between food restriction and controls (Table 3). Among matings to wild-type males, the ratio of females to males did not differ from the expected 1:1 (Table 3). The sex ratio tended to be male biased in controls and female-biased in food restriction pregnancies (*P* = 0.054, Table 3), in contrast to the results in the FCG matings.

**Table 3.** Fetal genotype ratios.

	Control	Food restriction	Total
	(N/%)	(N/%)	(N/%)
**FCG**			
XXF	31 (22.6%)	36 (24.8%)	67 (23.8%)
XXM	32 (23.4%)	37 (25.5%)	69 (24.5%
XYF	37 (27.0%)	32 (22.1%)	69 (24.5%)
XYM	37 (27.0%)	40 (27.6%)	77 (27.3%)
XX	63 (46.0%)	73 (50.3%)	136 (48.2%)
XY	74 (54.0%)	72 (49.7%)	146 (51.8%)
Gonadal female	68 (49.6%)	68 (46.9%)	136 (48.2%)
Gonadal male	69 (50.4%)	77 (53.1%)	146 (51.8%)
**Wild type**			
Female	36 (43.9%)	45 (59.2%)	81 (51.3%)
Male	46 (56.1%)	31 (40.8%)	77 (48.7%)

FCG, four core genotypes; XX, FCG animals with XX chromosomes, including both gonadal males and females; XXF, gonadal female with XX chromosomes; XXM, gonadal male with XX chromosomes; XY, FCG animals with XY chromosomes, including both gonadal males and females; XYF, gonadal female with XY chromosomes; XYM, gonadal male with XY chromosomes.

In no case did a chi-square test indicate that genotype ratios differed between treatments, or that genotype ratios differ from expected, i.e. from 1:1:1:1 for FCG or 1:1 for XX vs XY or gonadal females vs gonadal males. However, the difference in the proportions of wild-type males and females between treatments was marginally non-significant (*P* = 0.054).

**Figure 2.**
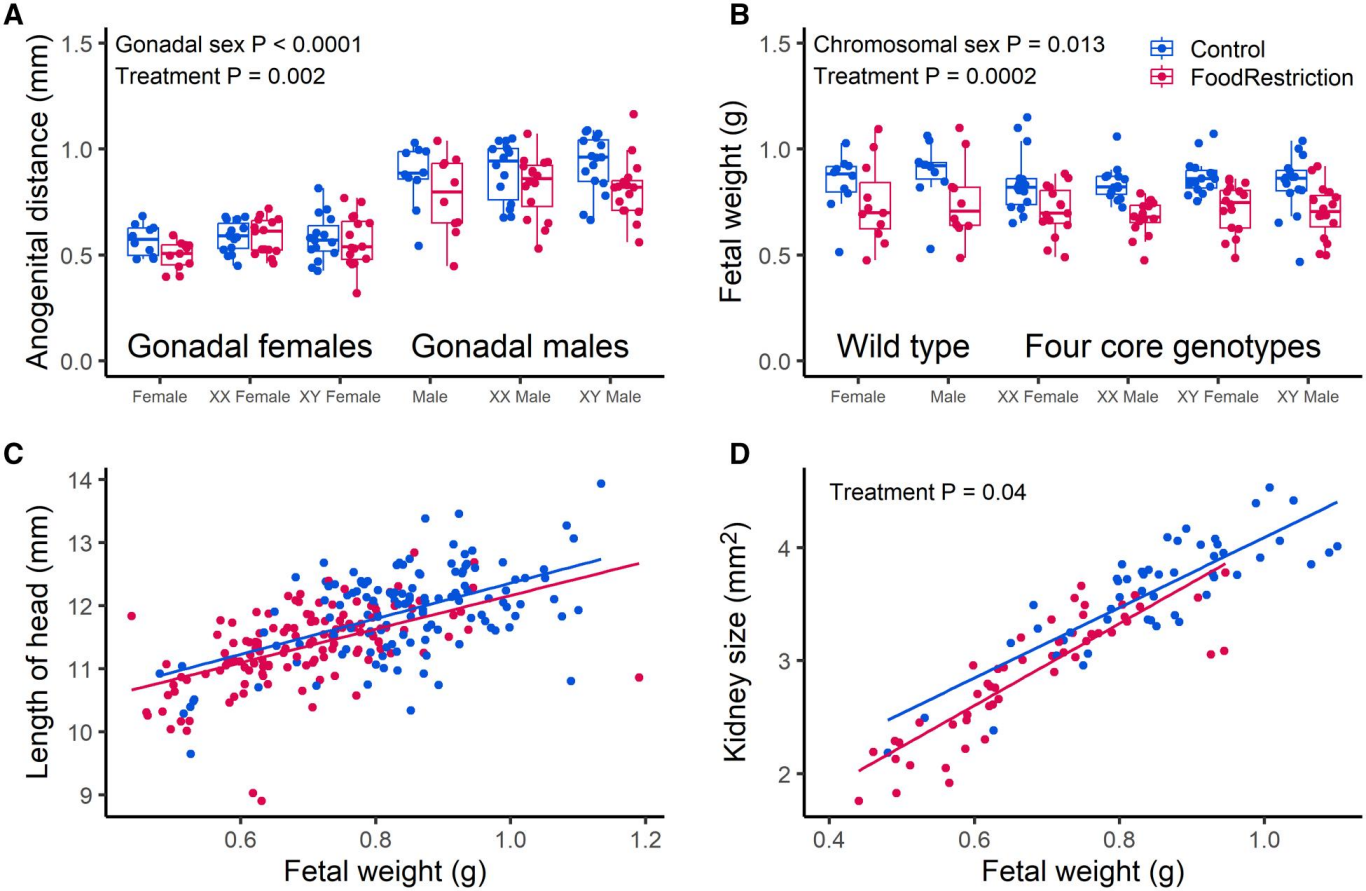
**Effects of treatment, gonadal sex, chromosomal sex, and mating type (four core genotypes (FCG) or wild type) on fetal traits.** Blue symbols: control; Red symbols: food restriction. (**A**) Anogenital distance (AGD). (**B**) Fetal weight. (**C**) Length of the fetal head. (**D**) Size of the fetal kidney. Plots A and B show average values per dam per genotype and plots C and D show individual fetuses, but all analyses described in text included all individual values and dam as a repeated, random subject. Only *P*-values for significant terms are shown in the figures; full models are described in the text.

To assess whether gonadal sex reflected exposure to gonadal hormones, we measured AGD, a known marker of prenatal androgen exposure (Hotchkiss and Vandenbergh, 2005). We fitted a model including effects of gonadal sex, chromosomal sex, treatment, mating type (FCG vs wild type), and the interaction between gonadal sex and mating type to test whether the difference between gonadal males and gonadal females was as great in FCG fetuses as in wild-type fetuses. The model included the dam as a repeated, random subject to account for non-independence among fetuses from the same dam. AGD was higher in gonadal XX and XY males than in gonadal XX and XY females (*F*_1, 52_=224.77, *P* < 0.0001; Fig. 2A), as expected. There was no effect of mating type on AGD (*F*_1, 52_=3.18, *P* = 0.08), no interaction between mating type and gonadal sex (*F*_1, 52_=0.49, *P* = 0.49), and no effect of chromosomal sex (*F*_1, 53_=0.01, *P* = 0.91). AGD was lower in fetuses exposed to food restriction (*F*_1, 52_=10.28, *P* = 0.002; Fig. 2A) reflecting a smaller size overall (as described in further detail below).

### Fetal weight and size: sex differences in response to food restriction?

To assess the effect of food restriction, and whether this depended on gonadal and/or chromosomal effect, we fitted a model including effects of gonadal sex, chromosomal sex, treatment, mating type (FCG vs wild type) and the interactions between gonadal sex and treatment and between chromosomal sex and treatment. As above, the model included the dam as a repeated, random subject to account for non-independence among fetuses from the same dam. Food-restricted fetuses were lighter than controls (*F*_1, 52_=15.49, *P* = 0.0002; Fig. 2B), but there were no interactions between gonadal sex and treatment (*F*_1, 52_=0.24, *P* = 0.63) or between chromosomal sex and treatment (*F*_1, 52_=0.01, *P* = 0.93). XY fetuses (including both gonadal males and females) were slightly heavier than XX fetuses (*F*_1, 52_=6.60, *P* = 0.013). Similar results were obtained for a linear measure of size, crown-rump length (effect of treatment: *F*_1, 52_=11.79, *P* = 0.001; Supplementary Fig. S1). To assess whether food restriction led to asymmetric growth restriction as a result of brain-sparing (Hiersch and Melamed, 2018), we analyzed head length with the model described above and fetal weight as a covariate. The effect of treatment was not significant (*F*_1, 48_=3.04, *P* = 0.09; Fig. 2C), indicating that food restriction fetuses did not have relatively large heads for their body weight, suggesting an absence of brain-sparing in either sex.

Because previous work identified sex-dependent effects of prenatal food restriction on adult blood pressure (Christians *et al.*, 2021), we assessed effects on the size of fetal kidneys. Fetal kidneys were smaller in food-restricted fetuses (*F*_1, 48_=32.33, *P* < 0.0001), but there were no effects of gonadal sex (*F*_1, 34_=1.15, *P* = 0.29) or chromosomal sex (*F*_1, 36_=0.82, *P* = 0.37), and no interactions between gonadal sex and treatment (*F*_1, 34_=0.01, *P* = 0.93) or between chromosomal sex and treatment (*F*_1, 36_=0.01, *P* = 0.94). The reduction in kidney weight in food restriction remained significant (*F*_1, 48_=4.46, *P* = 0.04) when fetal weight was added as a covariate, i.e. kidney size was reduced more than expected given the reduction in fetal weight (Fig. 2D).

### Placental weight, efficiency, and histology: sex differences in response to food restriction?

**Figure 3.**
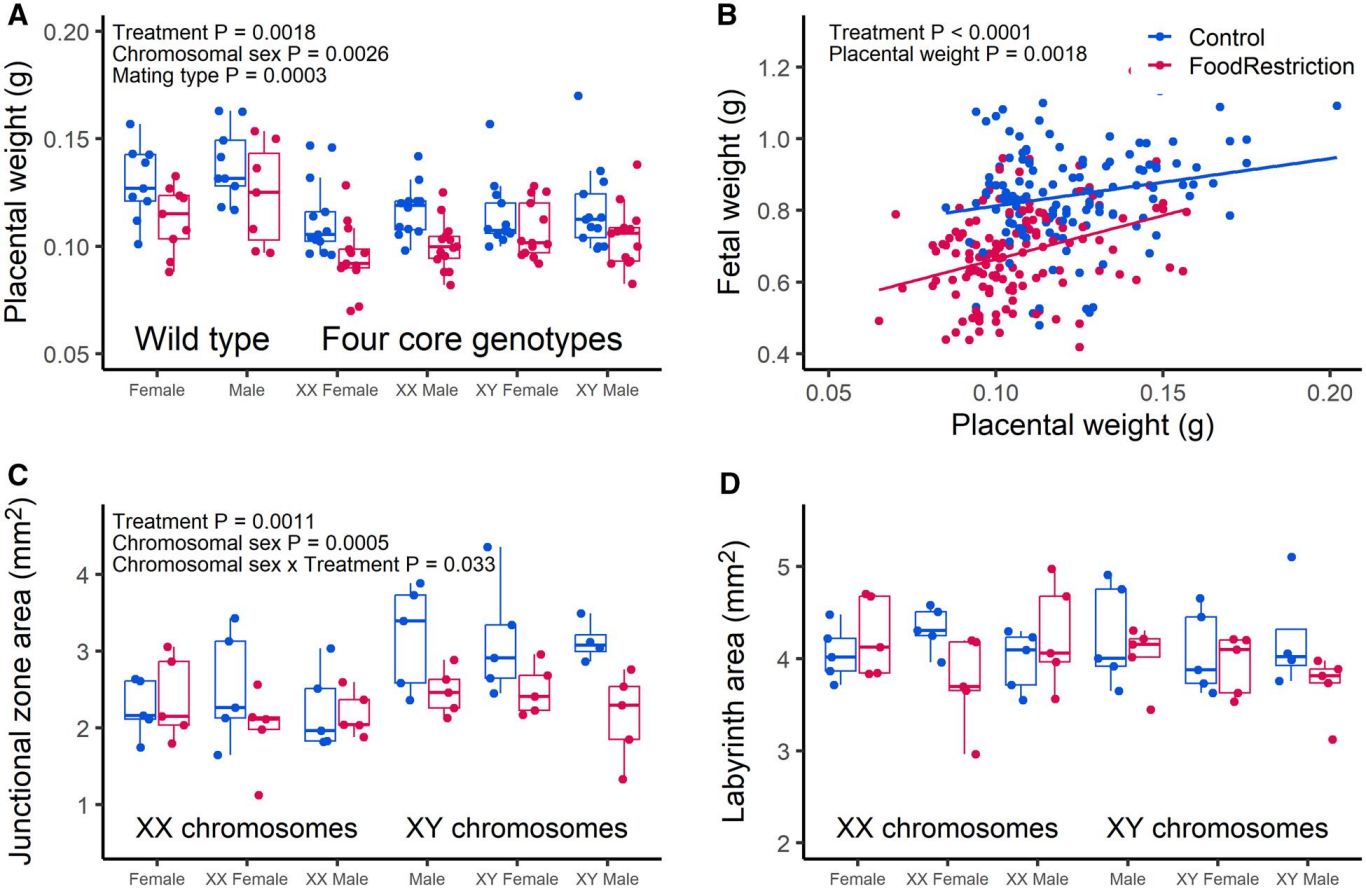
**Effects of treatment, gonadal sex, chromosomal sex, and mating type (four core genotypes (FCG) or wild type) on placental traits.** Blue symbols: control; Red symbols: food restriction. (**A**) Placental weight. (**B**) Relationship between placental weight and fetal weight. (**C**) Area of the junctional zone. (**D**) Area of the labyrinth. Plot A shows average values per dam per genotype, while plot B shows individual fetuses, but analyses described in text included all individual values and dam as a repeated, random subject. Only *P*-values for significant terms are shown in the figures; full models are described in the text. Plots C and D present average values per placenta.

The same analysis was performed for placental weight. The effect of treatment (*F*_1, 49_=10.88, *P* = 0.0018) was significant, with food-restricted placentas lighter than controls (Fig. 3A), but again there were no interactions between gonadal sex and treatment (*F*_1, 43_=0.22, *P* = 0.64) or between chromosomal sex and treatment (*F*_1, 45_=0.25, *P* = 0.62). Both gonadal male and female XY placentas were significantly heavier than XX placentas (*F*_1, 45_=10.15, *P* = 0.0026), but there was no effect of gonadal sex (*F*_1, 43_=1.26, *P* = 0.27; Fig. 3A). FCG placentas were lighter than wild-type placentas (*F*_1, 49_=15.58, *P* = 0.0003; Fig. 3A).

To assess placental efficiency while avoiding problems associated with the use of the ratio of fetal weight to placental weight (Christians *et al.*, 2018a), we repeated the analysis of fetal weight described above and included placental weight as a covariate. Food restriction fetuses remained smaller than controls, when controlling for placental weight (*F*_1, 49_=18.41, *P* < 0.0001; Fig. 3B), reflecting lower fetal weight for a given placental weight, i.e. lower placental efficiency.

We assessed the area of the labyrinth and the junctional zone in a subset of samples (N = 5 per genotype/treatment for a total of 60 placentas); representative images of placental histology are shown in Supplementary Fig. S2. The area of the junctional zone showed a significant interaction between chromosomal sex and treatment (*F*_1, 52_=4.80, *P* = 0.033), whereby XY control placentas had larger junctional zones than the other groups (Fig. 3C). In contrast, the area of the labyrinth did not show such an interaction (*F*_1, 52_=1.38, *P* = 0.25), or an effect of treatment (*F*_1, 52_=2.09, *P* = 0.15; Fig. 3D).

### Placental gene expression: sex differences in response to food restriction?

We aimed to analyze gene expression in only one placenta from each dam (sampling from the two placentas closest to one of the ovaries), but it was necessary to use two placentas of different genotypes from each of six dams to balance sample sizes (Table 1). In the labyrinth and in the junctional zone/decidua, 17 106 and 16 388 transcripts remained after pre-filtering, respectively. Across all samples, transcriptomic signatures were >96% similar in both tissues (Supplementary Fig. S3). For both tissues, we fitted a model in DESeq2 including gonadal sex, chromosomal sex, treatment, mating type, and the interactions between gonadal sex and treatment, and between chromosomal sex and treatment, as well as the number of unique reads in each sample identified by DRAGEN (scaled to a *z*-score), as this was associated with the first principal component of the count data. In both the labyrinth and the junctional zone/decidua, few genes showed an interaction between chromosomal sex and treatment, or an interaction between gonadal sex and treatment (Table 4).

**Table 4.** Number of differentially expressed genes.

	Labyrinth	Junctional zone/decidua
	Number of DEG	Number of DEG
**Treatment (food restriction vs control)**	525	665
**Chromosomal Sex (XX vs XY)**	30	18
**Gonadal sex (gonadal male vs female)**	0	0
**Mating type (C57BL/6J vs FCG)**	2	1
**Treatment × chromosomal sex interaction**	1	0
**Treatment × gonadal sex interaction**	0	1

DEG, differentially expressed genes; FCG, four core genotypes.

### Placental gene expression: effects of food restriction

**Figure 4.**
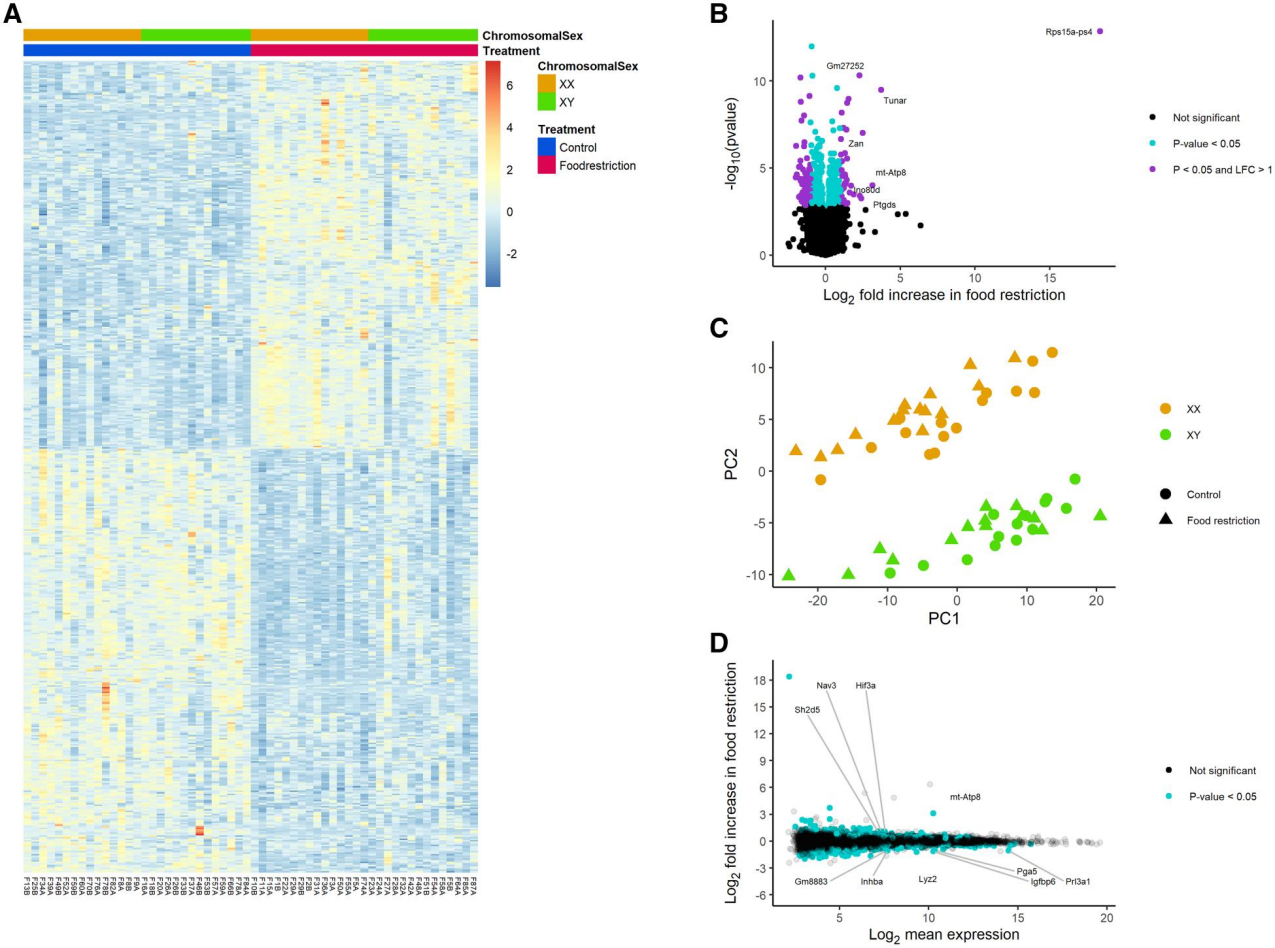
**Effects of food restriction on gene expression in the labyrinth.** (**A**) Heat map of genes differentially expressed. (**B**) Volcano plot; gene names are provided where the absolute log_2_-fold change was >2. (**C**) Principal component (PC) analysis of labyrinth samples, based on the top 500 most variable genes after variance stabilizing transformation (VST) by DESeq2. (**D**) MA plot; gene names are provided where the absolute log_2_-fold change was >1 and the log_2_ mean expression was >7.

**Figure 5.**
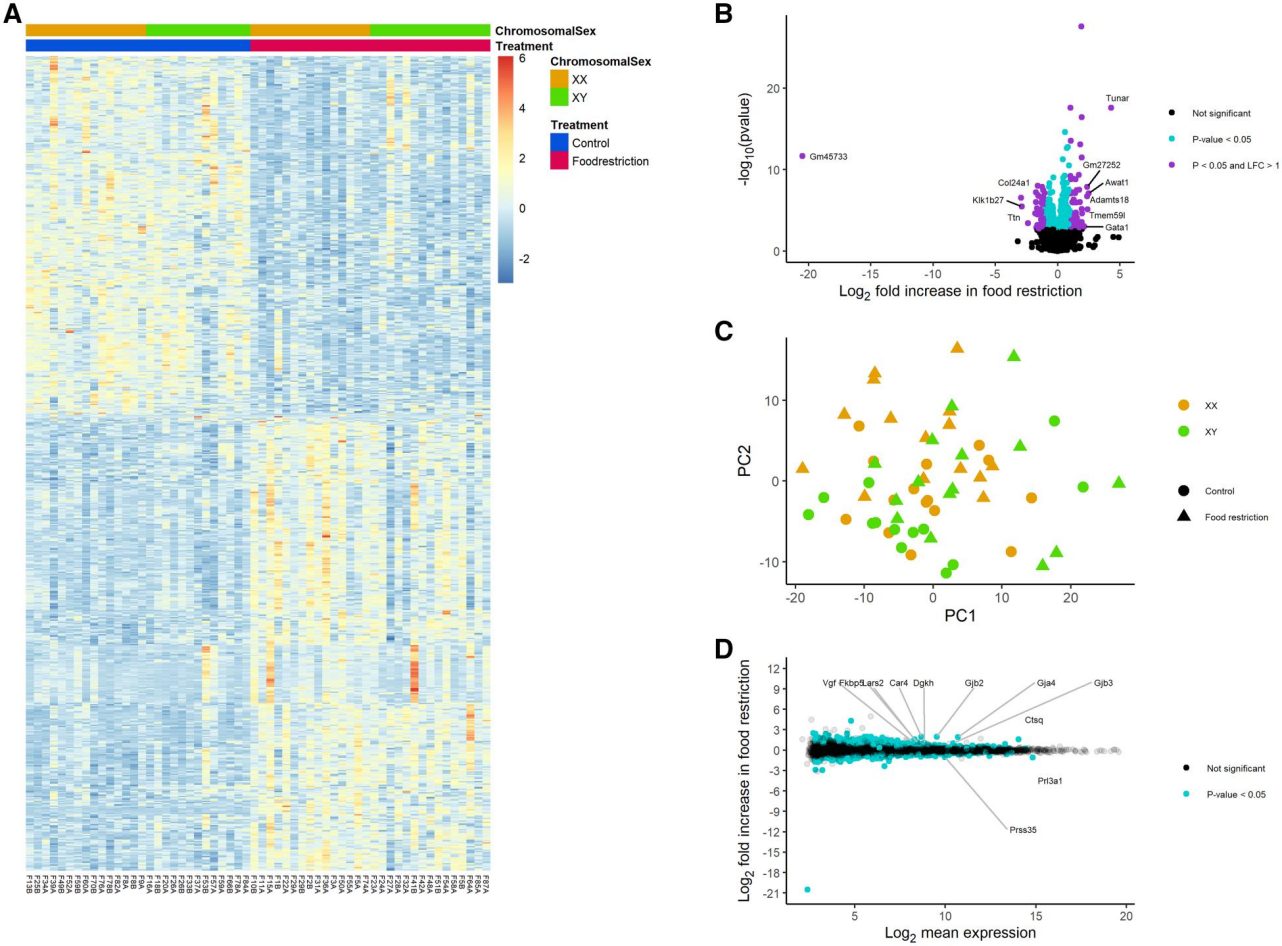
**Effects of food restriction on gene expression in the junctional zone/decidua.** (**A**) Heat map of genes differentially expressed. (**B**) Volcano plot; gene names are provided where the absolute log_2_-fold change was >2. (**C**) Principal component (PC) analysis of junctional zone/decidua, based on the top 500 most variable genes after variance stabilizing transformation (VST) by DESeq2. (**D**) MA plot; gene names are provided where the absolute log_2_-fold change was >1 and the log_2_ mean expression was >8.

**Figure 6.**
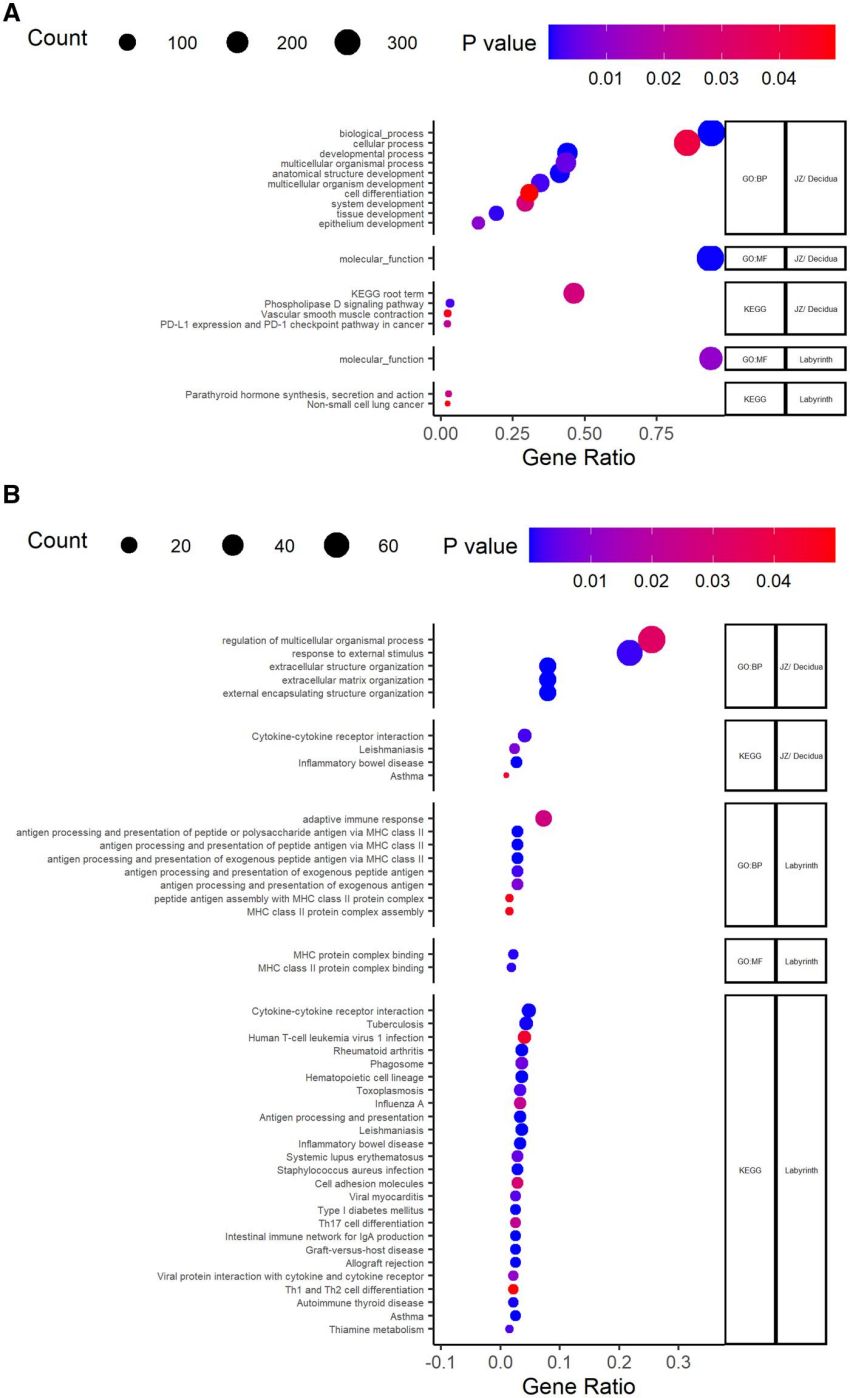
**Functional enrichment analysis of genes affected by food restriction.** (**A**) Genes upregulated and (**B**) genes downregulated by food restriction in the labyrinth and junctional zone/decidua, including gene ontology (GO) terms for biological processes (BP) and molecular functions (MF), as well as Kyoto Encyclopedia of Genes and Genomes (KEGG) pathways.

**Figure 7.**
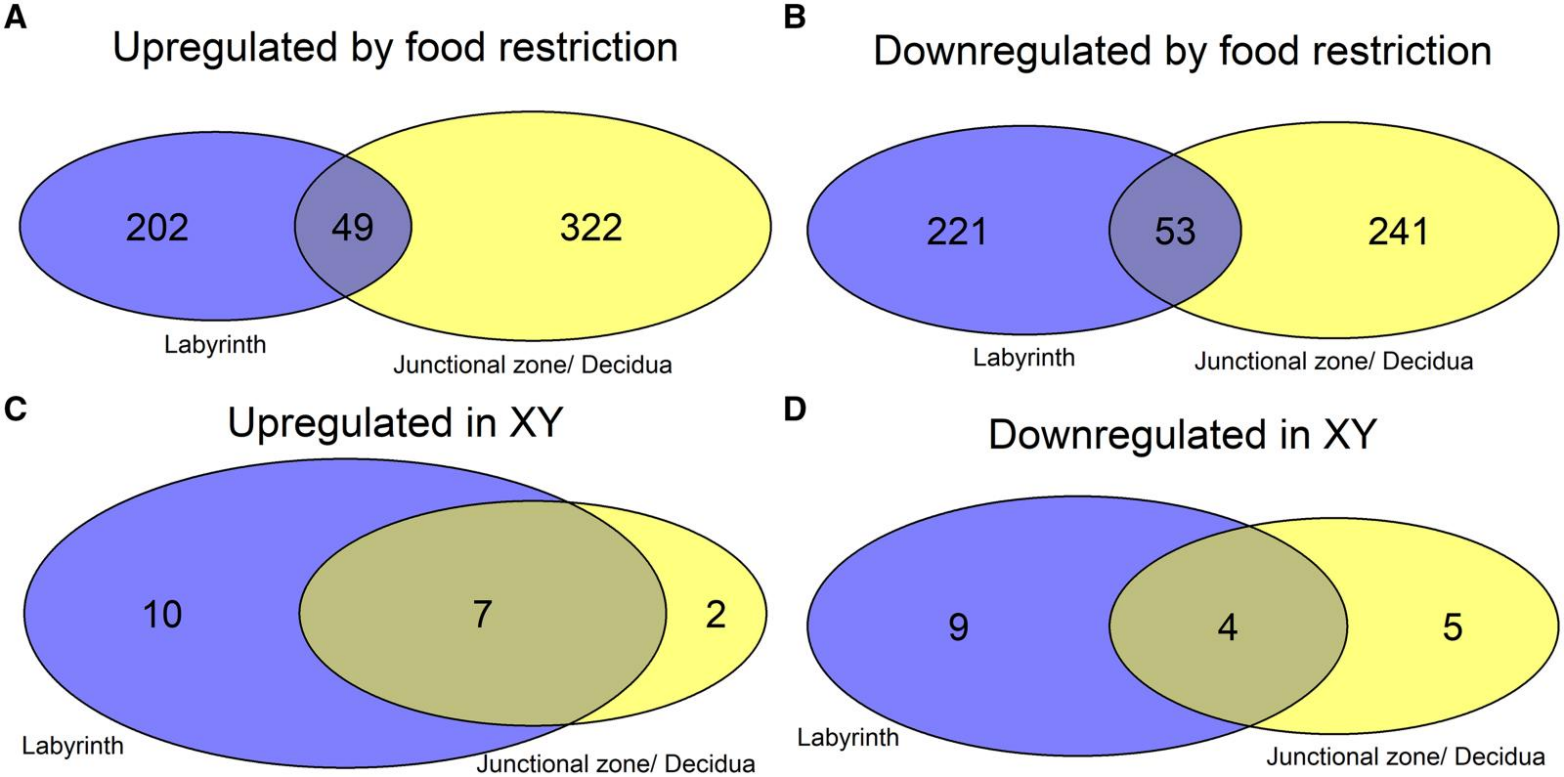
**Overlap in effects of food restriction or chromosomal sex between the labyrinth and junctional zone/decidua.** (**A**) Overlap in genes upregulated by food restriction. (**B**) Overlap in genes downregulated by food restriction. (**C**) Overlap in genes upregulated in XY tissues. (**D**) Overlap in genes downregulated in XY tissues.

Food restriction was associated with the upregulation of 251 and 371 genes in the labyrinth (Fig. 4A and B) and junctional zone/decidua (Fig. 5A and B), respectively (Table 4; Supplementary Tables S3 and S4). Among these genes, enriched gene ontology terms and KEGG pathways were very general (e.g. ‘molecular function’; Fig. 6A). In the labyrinth (Fig. 4A and B) and junctional zone/decidua (Fig. 5A and B), food restriction was associated with the downregulation of 274 and 294 genes, respectively (Table 4; Supplementary Tables S3 and S4). In both tissues, these genes were enriched for terms related to immune function (Fig. 6B). Despite some similarities in enriched pathways, relatively few of the genes affected by food restriction (<20%) were common to both the labyrinth and junctional zone/decidua (Fig. 7A and B). Nutrient transporters previously found to be affected by food restriction, such as *Slc2a1*, *Slc2a3*, *Slc38a1*, *Slc38a2*, *Slc38a4*, and *Got2* (*Fabppm*) (Coan *et al.*, 2010; Sferruzzi-Perri *et al.*, 2011; Ganguly *et al.*, 2014; Connor *et al.*, 2020; Eaton *et al.*, 2020; Van Gronigen Case *et al.*, 2021), and the stress-responsive *Ogt*, were not among genes differentially expressed by treatment in either tissue (Supplementary Tables S3 and S4). Despite the large number of genes differentially expressed by treatment, principal component analysis (PCA) plots did not show clear separation between control and food restriction samples (Figs 4C and 5C), likely because relatively few genes showed large changes in expression (Figs 4B and 5B). Furthermore, in both tissues, many differentially expressed genes had low expression levels, and large absolute log-fold changes (>4) were only seen in the genes with very low expression (Figs 4D and 5D).

### Placental gene expression: relative contributions of chromosomal and gonadal sex

**Figure 8.**
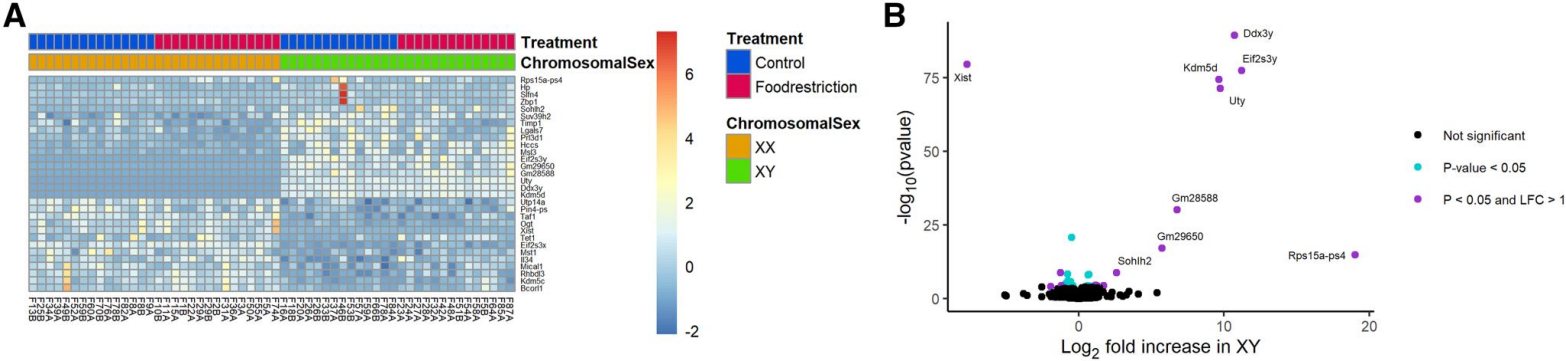
**Effects of chromosomal sex on gene expression in the labyrinth.** (**A**) Heat map of genes differentially expressed. (**B**) Volcano plot; gene names are provided where the absolute log_2_-fold change was >2.

**Figure 9.**
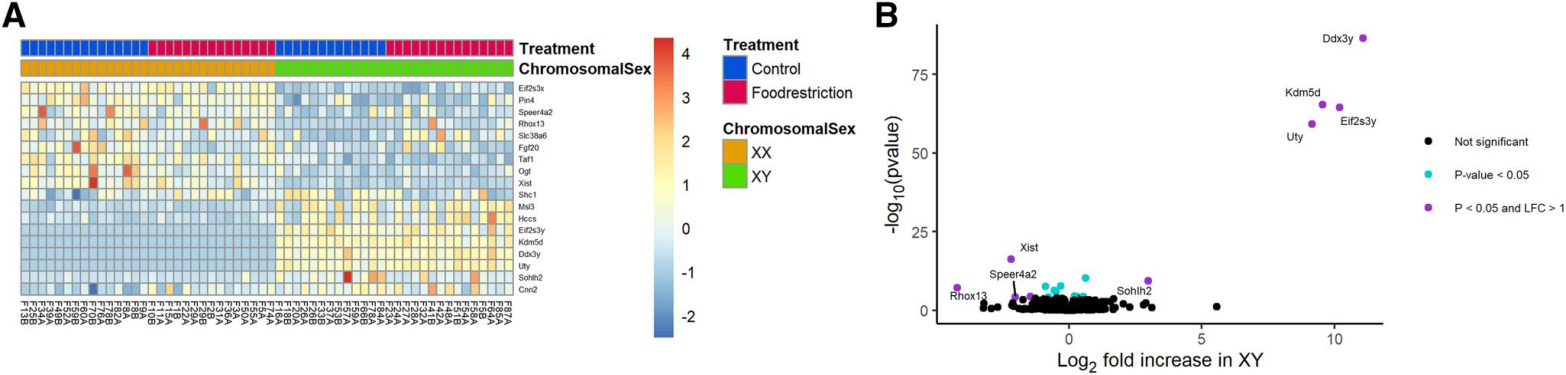
**Effects of chromosomal sex on gene expression in the junctional zone/decidua.** (**A**) Heat map of genes differentially expressed. (**B**) Volcano plot; gene names are provided where the absolute log_2_-fold change was >2.

No genes showed differential expression by gonadal sex (Table 4). In contrast, in the labyrinth (Fig. 8) and junctional zone/decidua (Fig. 9), 17 and 9 genes were upregulated in XY compared to XX, respectively, whereas 13 and 9 genes were downregulated in XY, respectively (Table 4; Supplementary Tables S5 and S6). Of these, 10 and 8 were on the X chromosome and 6 and 4 were on the Y chromosome in the labyrinth and junctional zone/decidua, respectively. Many of these genes were common to both the labyrinth and junctional zone/decidua (Fig. 7C and D); only one of the genes in common, *Sohlh2*, is not located on a sex chromosome. One X chromosome gene (*Eif2s3x*) and some of the Y chromosome genes (*Ddx3y*, *Kdm5d*, *Eif2s3y*, *Uty*) differentially expressed by chromosomal sex are members of homologous X–Y pairs that may act to maintain gene dosage (Bellott *et al.*, 2014). The gene encoding O-linked N-acetylglucosamine transferase (*Ogt*), which is known to escape X chromosome inactivation and be responsive to stress (Howerton *et al.*, 2013), was more highly expressed in XX mice in both tissues, as expected, but did not show an interaction between chromosomal sex and treatment. Two X-linked genes, *Msl3* and *Hccs*, were more highly expressed in XY mice, reflecting a known translocation from the X chromosome to the FCG Y^−^ chromosome (Panten *et al.*, 2024). In the labyrinth, XX and XY samples showed clear separation by PC2 (Fig. 4C), perhaps because of a few genes with large differences between XX and XY (Fig. 8), although such separation was not observed in the junctional zone/decidua (Fig. 5C).

### Placental gene expression: expression of sex steroid receptors

**Figure 10.**
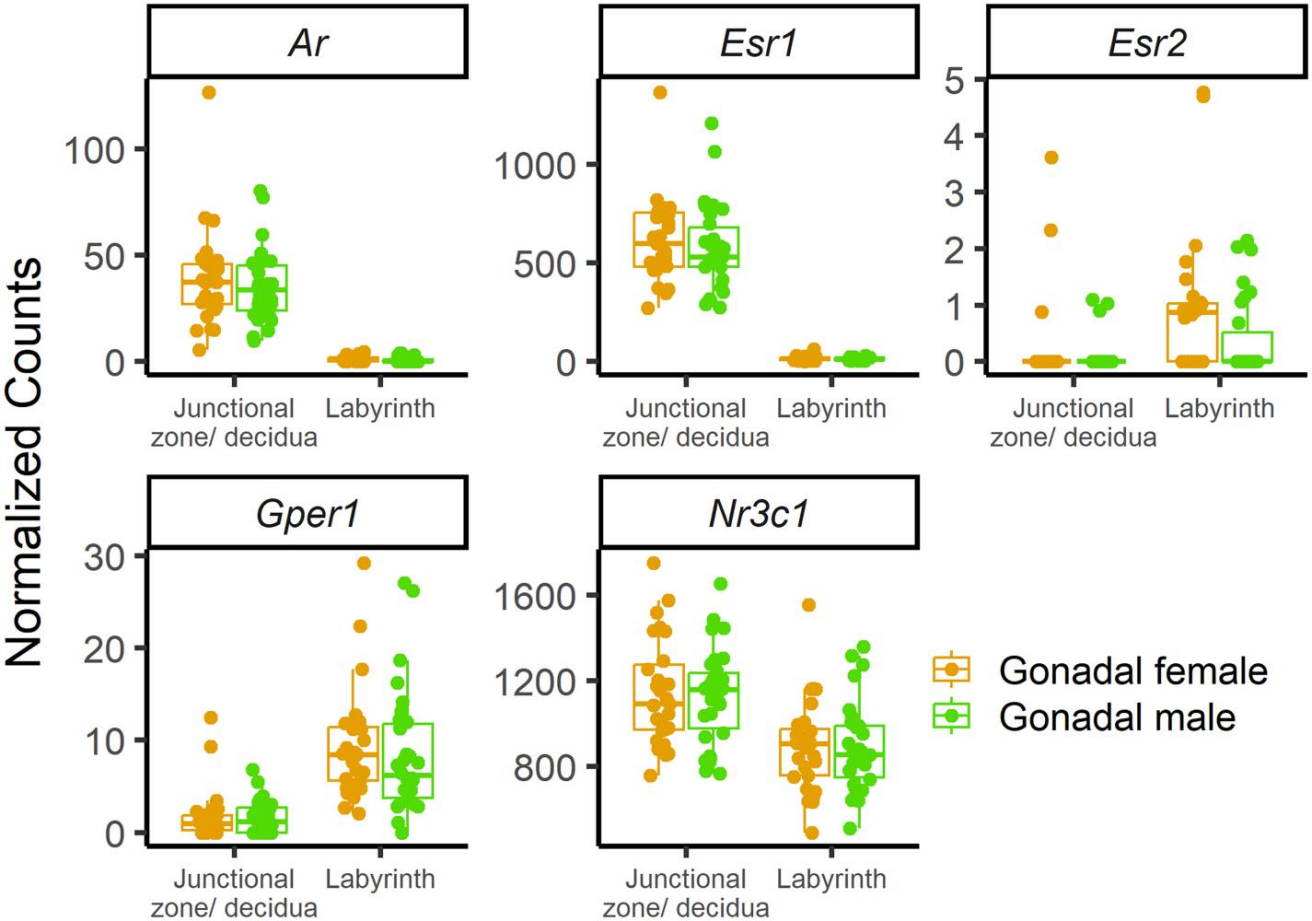
**Expression levels of the steroid hormone receptors.** Normalized counts of androgen receptor (*Ar*), estrogen receptor 1 (alpha) (*Esr1*), estrogen receptor 2 (beta) (*Esr2*), G protein-coupled estrogen receptor 1 (*Gper1*), and glucocorticoid receptor (*Nr3c1*) in the labyrinth and junctional zone/decidua.

Given the lack of effects of gonadal sex on gene expression, we examined expression levels of the sex steroid receptors. In the labyrinth, the expression of the androgen receptor (*Ar*), estrogen receptor 1 (alpha) (*Esr1*), estrogen receptor 2 (beta) (*Esr2*), and G protein-coupled estrogen receptor 1 (*Gper1*) was either very low or not detected (Fig. 10), whereas in the junctional zone/decidua samples, there was moderate expression of *Esr1* and low expression of *Ar* (Fig. 10). The glucocorticoid receptor (*Nr3c1*), for which binding sites are enriched among genes affected by sex steroids in mouse liver and adipose tissue (Blencowe *et al.*, 2022), was highly expressed in both the labyrinth and the junctional zone/decidua (Fig. 10).

### Validation of effects of treatment and chromosomal sex on placental gene expression

**Figure 11.**
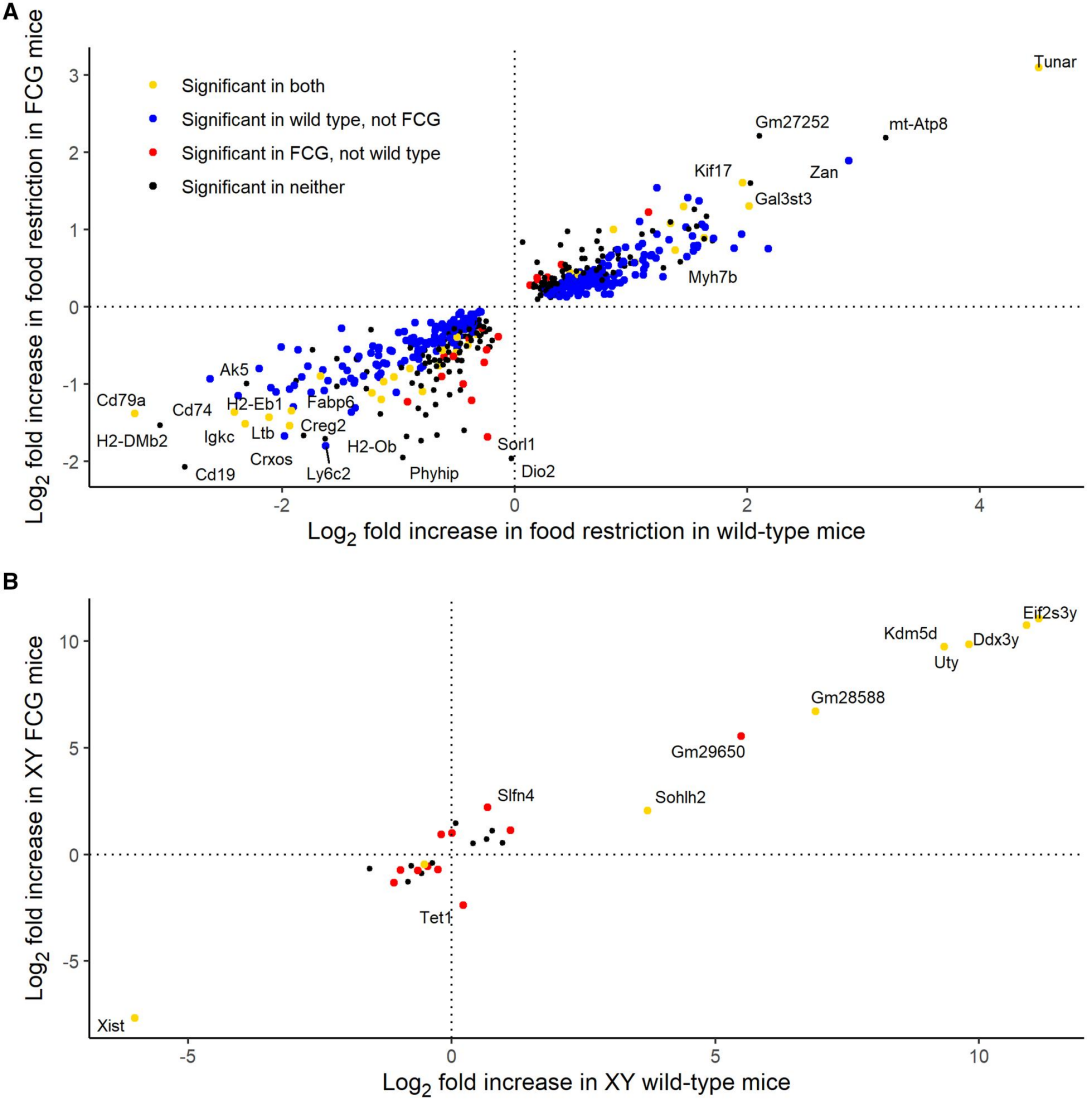
**Consistency of effects of treatment and chromosomal sex in the labyrinth when four core genotypes (FCG) and wild-type samples were analyzed separately.** (**A**) Effects of treatment. (**B**) Effects of chromosomal sex. Only genes showing significant differential expression in the combined analysis are included. Symbol color indicates whether effect was significant in both FCG and wild-type sub-analyses (yellow), significant in wild type but not FCG (blue), significant in FCG but not wild type (red), or not significant in either of the sub-analyses, but significant in the combined analysis (black). Gene names are provided where the absolute log_2_-fold change in the combined analysis was >1.5.

**Figure 12.**
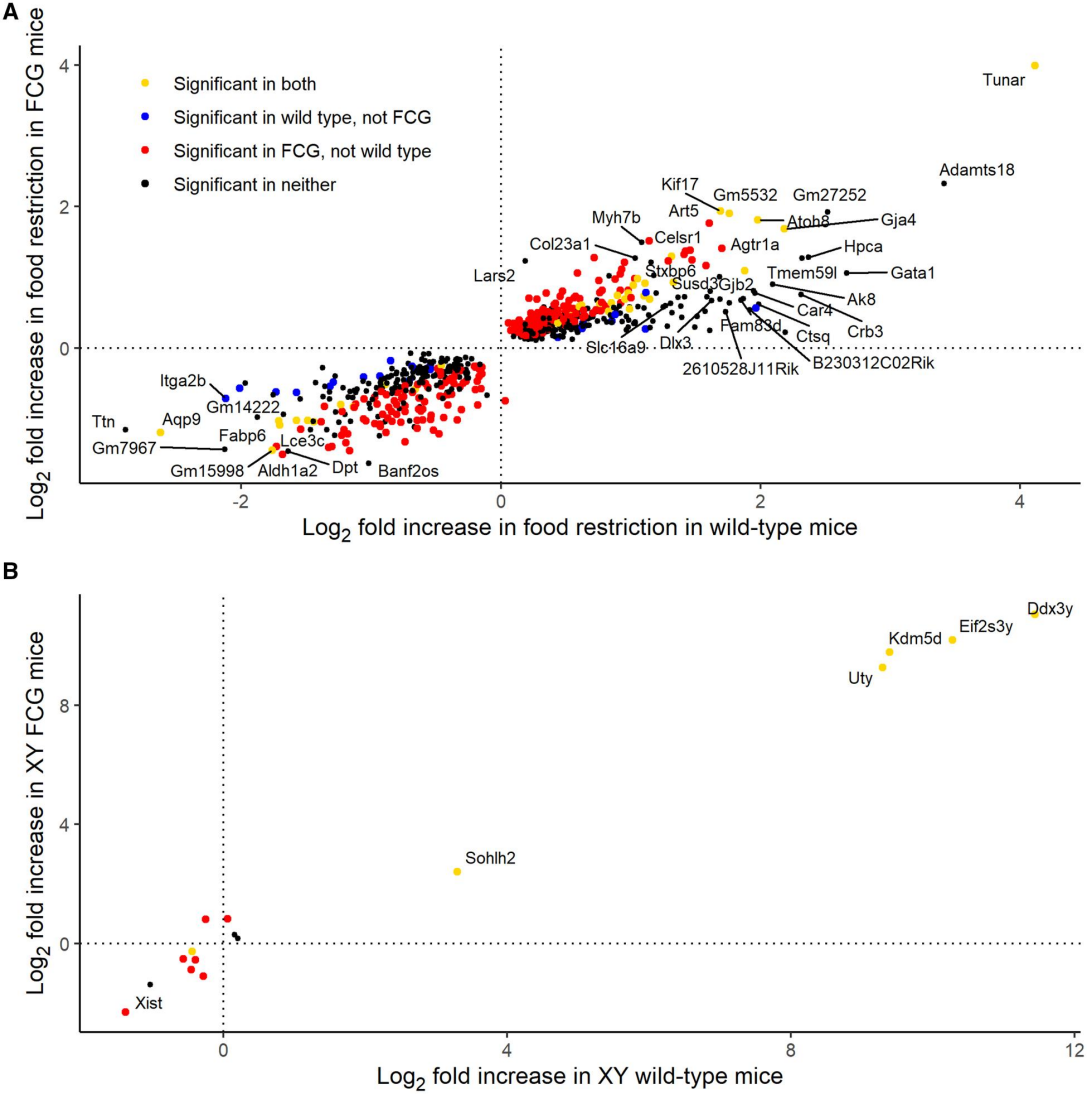
**Consistency of effects of treatment and chromosomal sex in the junctional zone/decidua when four core genotypes (FCG) and wild-type samples were analyzed separately.** (**A**) Effects of treatment. (**B**) Effects of chromosomal sex. Only genes showing significant differential expression in the combined analysis are included. Symbol color indicates whether effect was significant in both FCG and wild-type sub-analyses (yellow), significant in wild type but not FCG (blue), significant in FCG but not wild type (red), or not significant in either of the sub-analyses, but significant in the combined analysis (black). Gene names are provided where the absolute log_2_-fold change in the combined analysis was >1.5.

To validate our DEG results and the combined analyses of FCG and wild-type samples, we analyzed samples sired by FCG and wild-type males separately. Among genes differentially expressed in the combined analyses, the log_2_-fold changes in FCG samples were strongly correlated with the log_2_-fold changes in wild-type samples for both the effects of treatment and chromosomal sex, in both the labyrinth and the junctional zone/decidua (Figs 11 and 12), i.e. the results were largely consistent in two independent sets of samples.

### Sex-stratified analyses of placental gene expression

**Figure 13.**
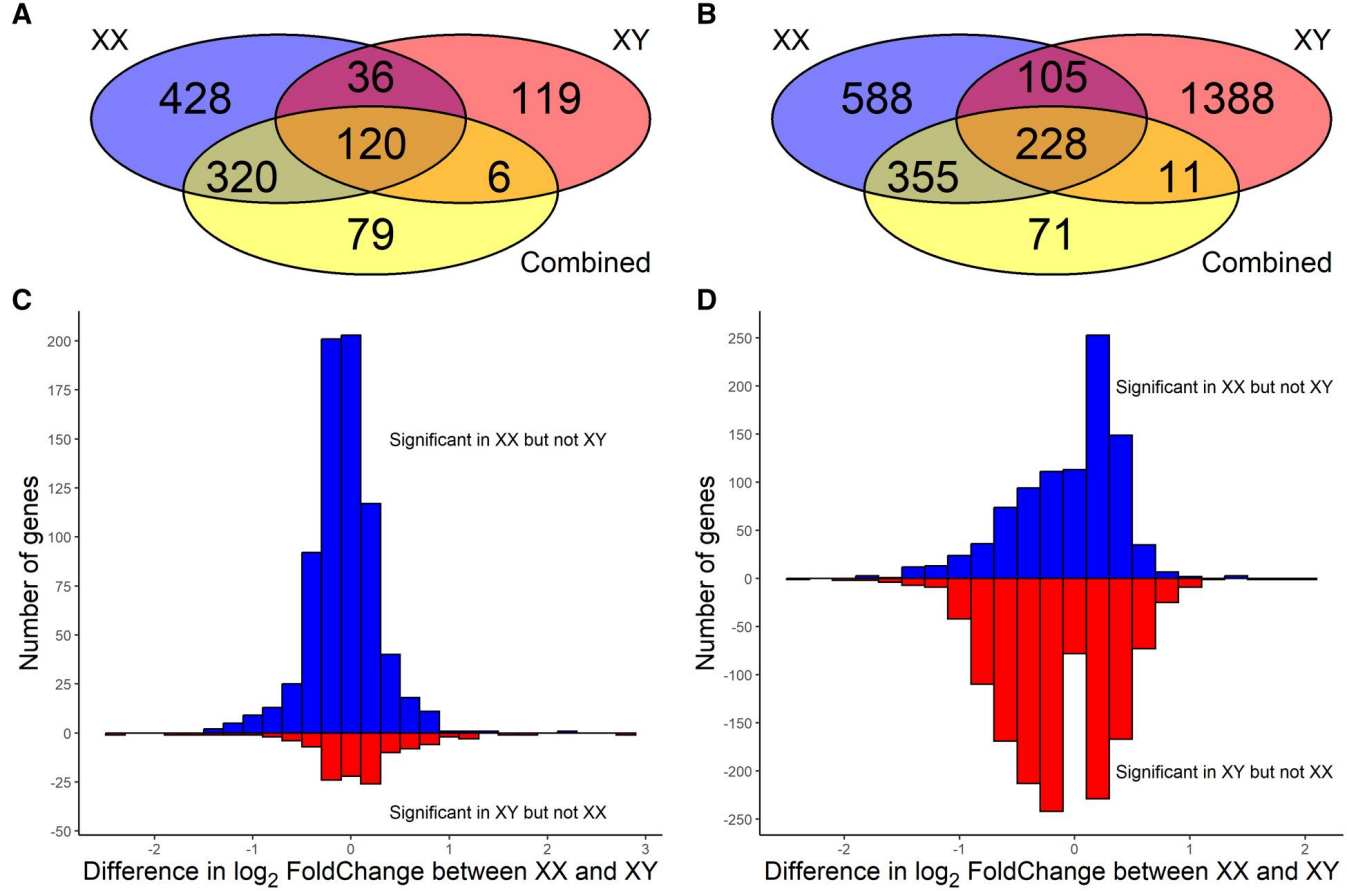
**Effects of treatment on placental gene expression from analyses stratified by chromosomal sex, including both four core genotypes (FCG) and wild-type samples.** (**A**) Venn diagram of the overlap in genes significantly affected by treatment (food restriction vs control) in the labyrinth in separate analyses for each chromosomal sex and in the combined analysis including both XX and XY. (**B**) Overlap in genes significantly affected by treatment in the junctional zone/decidua. (**C**) Distribution of the difference in estimated effect of treatment (log_2_-fold change) in the labyrinth between XX and XY placentas among genes significant in XX but not XY placentas (above, blue), and vice versa (below, red). (**D**) Distribution of the difference in estimated effect of treatment between the chromosomal sexes in the junctional zone/decidua.

Our finding that there were no interactions between treatment and gonadal or chromosomal sex is at odds with many previous studies reporting numerous sex-specific effects. However, many previous studies have analyzed the sexes separately, rather than explicitly testing whether effects of treatment were significantly different between the sexes, and this may inflate the rate of false positives (Chin and Christians, 2015; Christians *et al.*, 2021; Christians and Chow, 2022). To investigate this possibility, we repeated our analyses separately by chromosomal sex, since we found more differences in gene expression by chromosomal sex than by gonadal sex. In both tissues, the sex-stratified analyses found hundreds of genes to be differentially expressed in response to treatment in one sex but not the other, including hundreds of genes that were not found to be significant in the combined analyses (Fig. 13A and B). These ‘sex-specific’ effects could be false positives or, alternatively, could be due to insufficient power to detect real interactions between sex and treatment. To assess this, we examined the distribution of the difference in log_2_-fold changes between XX and XY samples and found that these were generally quite close to zero (Fig. 13C and D), indicating that, even if some of the ‘sex-specific’ effects are real, there are few genes with a substantially different response in males and females.

## Discussion

### Sex differences in fetal and placental responses to food restriction

Our first goal was to test the hypothesis that males prioritize fetal growth at the expense of placental development, whereas females are better able to respond to prenatal adversity. Our model of prenatal adversity, maternal food restriction beginning in mid-gestation, reduced maternal weight, fetal weight, and size as well as placental weight, without affecting the number of fetuses that appeared viable. Despite treatment itself having clear effects, the effects on fetal and placental weights did not depend on gonadal or chromosomal sex. In our previous systematic review and meta-analysis (Christians *et al.*, 2021), we similarly found that food restriction in the latter half of gestation in rats consistently reduced birthweight, but that these effects did not differ between the sexes. Undernutrition also affects fetal growth in both sexes in the mouse (Goyal *et al.*, 2013; Eaton *et al.*, 2020). The only consistent sex-dependent effect of prenatal food restriction that we observed in our previous review was an increase in adult blood pressure, with males affected to a greater extent than females (Christians *et al.*, 2021). Given this latter observation, in the present study, we examined fetal kidney size to test the hypothesis that kidney development and nephron complement underlie the effects of food restriction on blood pressure (Morton *et al.*, 2016). We observed that fetal kidney size was disproportionately reduced by food restriction, i.e. to a greater extent than expected given the effect on fetal weight, but this effect was not sex dependent.

In contrast, the junctional zone area of XY placentas was more responsive to food restriction than it was in XX placentas, with larger junctional zones in XY control placentas, and no difference between the sexes in food restriction. This finding is inconsistent with the hypothesis that males prioritize fetal over placental growth and are less able to respond to insults. Previous work has similarly found maternal protein restriction to reduce the junctional zone but not the labyrinth (Sferruzzi-Perri *et al.*, 2011; Gonzalez *et al.*, 2016), and found junctional zone areas to be smaller in female placentas than in male placentas among controls (Eaton *et al.*, 2020).

Few genes showed interactions between treatment and gonadal or chromosomal sex in expression levels. We thus found little support for the hypothesized sex differences in fetal growth strategies. While some authors report extensive sex-specific effects in the placenta, rather than testing for interactions between sex and treatment, many such studies analyze the sexes separately, which is an approach that can greatly inflate the number of false positives (Christians *et al.*, 2021; Christians and Chow, 2022). This was illustrated by our sex-stratified analyses, which found hundreds of genes to be significantly affected by food restriction in one sex but not the other, even though differences in effect size between the sexes (measured as log_2_-fold change) were generally small.

### Relative contributions of chromosomal and gonadal sex

Our second goal was to use the FCG mouse model to assess the relative contributions of chromosomal complement and fetal gonads to sex differences in placental gene expression, which have not been investigated previously. Genotype ratios did not vary from expected, and the number of conceptuses did not vary between FCG and wild-type matings, suggesting no effect on survival in the model. AGD, a known marker of prenatal androgen exposure (Hotchkiss and Vandenbergh, 2005), varied with the gonadal sex of the fetuses and suggested that sex differences in gonadal hormone levels in the FCG fetuses reflected those in wild-type fetuses. This result has previously been reported in FCG mice at 4 weeks of age (Itoh *et al.*, 2015), but not prenatally. However, a potential caveat regarding the use of the FCG model was that placental weights were lower in FCG pregnancies than in wild-type pregnancies, even in XX female conceptuses, which are expected to be genetically identical in FCG and wild-type matings. Pregnancies sired by FCG and wild-type males involved the same cohort of females and occurred at the same time, indicating that differences in placental weight were due to the males. While FCG and wild-type males shared a C57BL/6J background, it is possible that the Y chromosome deletion and/or *Sry* transgene affected spermatogenesis leading to epigenetic differences in sperm that affected placental weight. These results illustrate that, while the FCG represents a powerful model for investigating the mechanisms underlying sex differences, it is important to include wild-type controls to assess the extent to which FCG mice reflect wild-type sex differences.

We found little to no effect of gonadal sex on fetal growth, placental growth, or placental gene expression, even though androgen levels differed by gonadal sex, as assessed by AGD. The lack of effect of gonadal sex on placental gene expression is unlikely due to low statistical power given the unusually large size of our RNAseq dataset, which included 28–30 biological replicates per gonadal sex per tissue. Our result regarding gonadal sex is consistent with the finding that sexual dimorphism in placental gene expression does not increase following fetal androgen production in humans (Braun *et al.*, 2021). We expected the placenta to be influenced by the gonadal status of the fetus, given that fetal sex can influence neighboring fetuses (vom Saal *et al.*, 1983; Hotchkiss and Vandenbergh, 2005), and the labyrinth is directly exposed to the fetal circulation. Androgen receptors are present in the human placenta (Meakin and Clifton, 2019; Meakin *et al.*, 2019), although we found the transcript count to be low in the junctional zone/decidua, and undetectable in the labyrinth. Even in the absence of sex steroid receptors, the placenta would be expected to respond to other sexually differentiated signals.

In contrast, XY fetuses and placentas were heavier than their XX counterparts, as previously observed (Ishikawa *et al.*, 2003). Approximately half of the genes showing expression differences between XX and XY placentas were located on either the X or Y chromosome, and some of these were members of homologous X–Y pairs thought to maintain gene dosage (Bellott *et al.*, 2014), i.e. despite showing differential expression, their effect may have been to reduce differences between the sexes.

Given its role in sex-dependent responses to maternal stress (Howerton and Bale, 2014; Bale, 2016), we expected that the X-linked gene *Ogt* might show a sex-dependent response to maternal nutrition. However, while *Ogt* expression was higher in XX than in XY placentas, as expected, it was not affected by food restriction. While a previous study found placental *Ogt* to be upregulated by a high-fat diet and a low-fat diet (compared to an intermediate control) (Mao *et al.*, 2010), other studies have not observed a response to diet (Gabory *et al.*, 2012; de Barros *et al.*, 2020).

### Effects of food restriction

As expected, food restriction reduced fetal weight near term, as has previously been observed in mice (Jimenez-Chillaron *et al.*, 2005; Coan *et al.*, 2010; Sferruzzi-Perri *et al.*, 2011; Ganguly *et al.*, 2016; Connor *et al.*, 2020) and rats (Christians *et al.*, 2021). Although we expected nutrient transporter genes to be responsive to food restriction, this was not observed. A less severe degree of food restriction beginning earlier in pregnancy increased the expression of system A amino acid transporter gene *Slc38a2* (Sferruzzi-Perri *et al.*, 2011) and the facilitated glucose transporter gene *Slc2a1* (Coan *et al.*, 2010), whereas a slightly more severe degree of food restriction decreased the expression of the glucose transporter *Slc2a3* (Ganguly *et al.*, 2014). Plasma membrane fatty acid binding protein, *Fabppm/Got2*, was also previously found to be increased by food restriction (Connor *et al.*, 2020). These previous studies took a targeted, candidate-gene approach in contrast to the transcriptome-wide approach used in the present study.

## Conclusions

We did not find evidence of the hypothesized sex differences in fetal growth strategy or their mediation by the placenta (Eriksson *et al.*, 2010; Meakin *et al.*, 2021). Despite the popularity of this hypothesis, it is often not supported by specific tests or systematic reviews in humans or animal models (Christians *et al.*, 2021, 2023; Christians and Chow, 2022; Hercus *et al.*, 2024a). While it is clear that males are overrepresented among adverse pregnancy outcomes, the sex ratio at conception is not different from 50:50, with female-biased mortality early in gestation (Orzack *et al.*, 2015). Thus, the increased frequency of complications among males later in pregnancy may reflect a selection bias, whereby female pregnancies with impaired placentation are lost early, and so are less likely to be included in studies of adverse outcomes such as premature birth and stillbirth.

Sex differences in placental gene expression were largely due to chromosomal sex and not to the gonadal sex of the fetus. Unraveling the mechanisms underlying sex differences will require animal models such as the one we have used. Nevertheless, a primary role for the sex chromosomes may also hold true in humans given that the initiation of fetal androgen production by human male fetuses does not seem to affect sex differences in the placenta (Braun *et al.*, 2021).

## Supplementary Material

gaaf015_Supplementary_Data

## Data Availability

Sequencing data are deposited in the GEO database under accession numbers GSE285453 (labyrinth) and GSE291544 (junctional zone/decidua).
