# Supplementary material for: Roles of chromosomal and gonadal sex in the fetal and placental responses to maternal food restriction in mice
Source: Mol Hum Reprod. 2025 Apr 26;31(2):gaaf015. doi: 10.1093/molehr/gaaf015 (PMC12085225; doi:10.1093/molehr/gaaf015)
Supplement: gaaf015_Supplementary_Data [file gaaf015_supplementary_data.zip › Supplementary Information Revised.pdf]

Roles of chromosomal and gonadal sex in the fetal and placental responses to maternal food restriction in mice

Jess C. Hercus, Daniel Alejandro Salcedo Rubio, Maria Elisa Osorio Nieto, Cheayeong Keum, Qi Wang, John A. Macdonald, Jordan S. Scott, Emily R. J. Lucas, and Julian K. Christians

Supplementary Information Contents

Supplementary Figure S1. Effects of treatment on fetal crown-rump length.

Supplementary Figure S2. Representative placental histology images.

Supplementary Figure S3. Sample-sample correlations.

Supplementary Tables (available as separate files)

Supplementary Table S1. RNA quality and sequencing metrics for labyrinth samples.

Supplementary Table S2. RNA quality and sequencing metrics for junctional zone/ decidua samples.

Supplementary Table S3. Effects of food restriction on gene expression in the labyrinth; a positive $\log_2$FoldChange indicates higher expression in food restricted placentas.

Supplementary Table S4. Effects of food restriction on gene expression in the junctional zone/ decidua; a positive $\log_2$FoldChange indicates higher expression in food restricted placentas.

Supplementary Table S5. Effects of chromosomal sex on gene expression in the labyrinth; a positive $\log_2$FoldChange indicates higher expression in XY placentas.

Supplementary Table S6. Effects of chromosomal sex on gene expression in the junctional zone/ decidua; a positive $\log_2$FoldChange indicates higher expression in XY placentas.

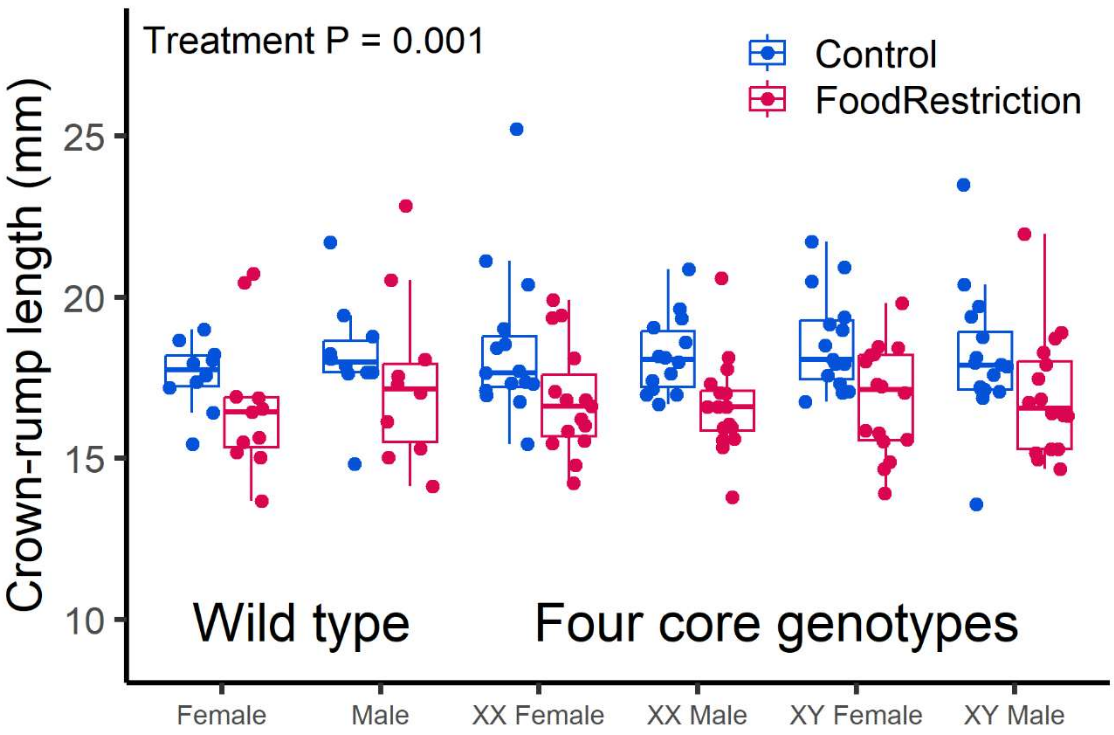

Supplementary Figure S1. Effects of treatment (blue symbols: control; red symbols: food restriction), gonadal sex, chromosomal sex, and mating type (FCG or wild type) on fetal crown-rump length. Plot shows average values per dam per genotype, but analysis described in text included all individual values and dam as a repeated, random subject. Only P values for significant terms are shown; full models are described in the text.

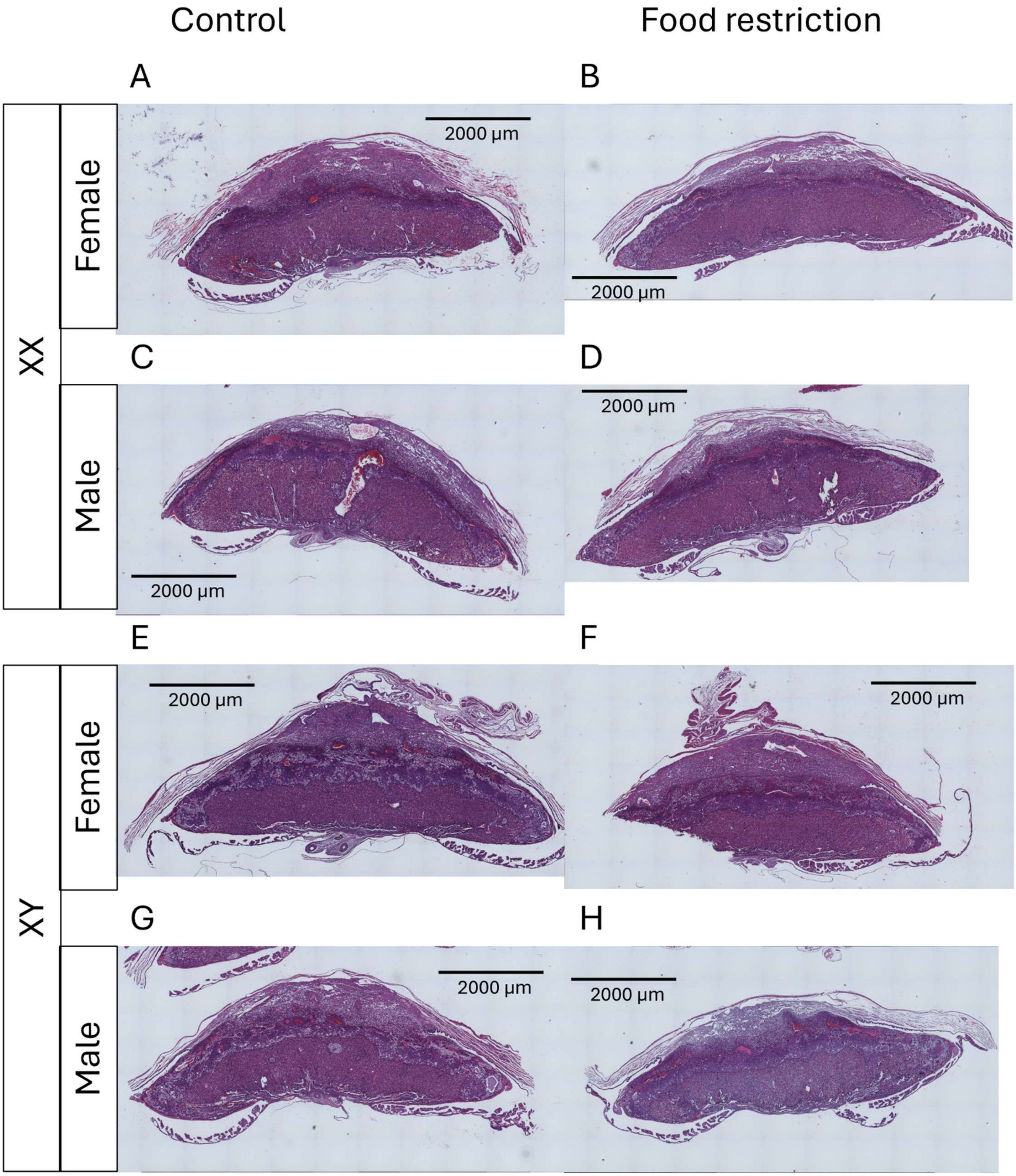

Supplementary Figure S2. Representative placental histology images. Samples (A) F16F, (B) F15D, (C) F26H, (D) F28D, (E) F25E, (F) F29F, (G) F13E, (H) F1F.

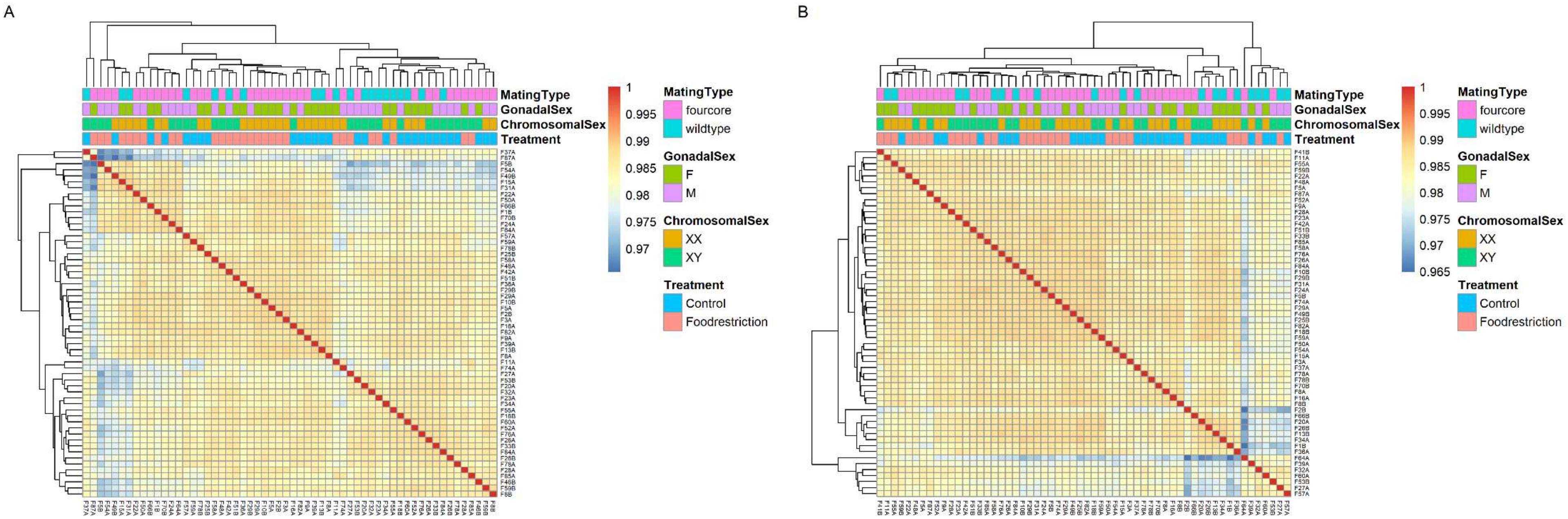

Supplementary Figure S3. Sample-sample correlations. (A) Labyrinth, (B) Junctional zone/ decidua.
