# Supplementary figures and images for: Roles of chromosomal and gonadal sex in the fetal and placental responses to maternal food restriction in mice
Source: Mol Hum Reprod. 2025 Apr 26;31(2):gaaf015. doi: 10.1093/molehr/gaaf015 (PMC12085225; doi:10.1093/molehr/gaaf015)

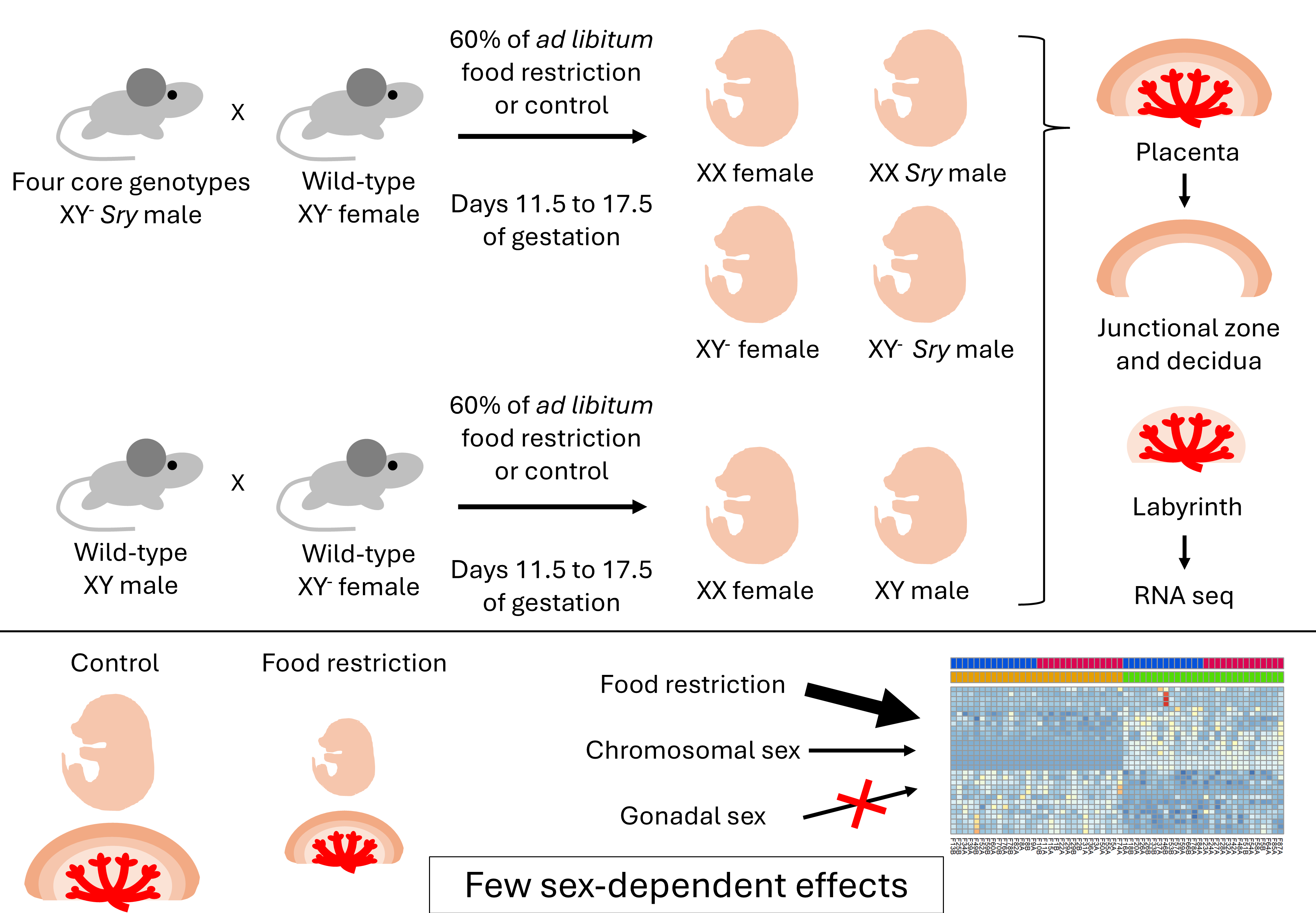

Supplement: gaaf015_Supplementary_Data [file gaaf015_supplementary_data.zip › Graphical Abstract.tif]
